# **Multi-omics evaluation of peritoneal fluid in gastroesophageal cancer (OMEGCA): protocol** for a **prospective multicentre cohort study to detect occult peritoneal metastases in patients undergoing curative-intent treatment**

**DOI:** 10.1371/journal.pone.0318615

**Published:** 2025-04-16

**Authors:** David S. Liu, Zexi Allan, Jeanne Tie, Katheryn Hall, Margaret M. Lee, Darren J. Wong, Stephen Q. Wong, Niall C. Tebbutt, David I. Watson, Markus Trochsler, Krinal Mori, Nicole Winter, Sarah Martin, Geraldine Ooi, Yahya Al-Habbal, Ronald Ma, Nicholas J. Clemons

**Affiliations:** 1 Division of Cancer Surgery, Peter MacCallum Cancer Centre, Melbourne, Victoria, Australia; 2 Division of Cancer Research, Peter MacCallum Cancer Centre, Melbourne, Victoria, Australia; 3 Upper Gastrointestinal Surgery Unit, Division of Surgery, Anaesthesia and Procedural Medicine, Austin Hospital, Heidelberg, Victoria, Australia; 4 Victorian Interventional Research and Trials Unit, Department of Surgery, University of Melbourne, Austin Precinct, Heidelberg, Victoria, Australia; 5 Department of Medical Oncology, Peter MacCallum Cancer Centre, Melbourne, Victoria, Australia; 6 Sir Peter MacCallum Department of Oncology, University of Melbourne, Parkville, Victoria, Australia; 7 Department of Medical Oncology, Eastern Health, Box Hill, Victoria, Australia; 8 Department of Gastroenterology, Division of Surgery, Anaesthesia and Procedural Medicine, Austin Hospital, Heidelberg, Victoria, Australia; 9 Department of Medical Oncology, Olivia Newton-John Cancer and Wellness Centre, Heidelberg, Victoria, Australia; 10 Oesophago-gastric Surgery Unit, Flinders Medical Centre, Bedford Park, South Australia, Australia; 11 Flinders Health and Medical Research Institute, Flinders University, Adelaide, South Australia, Australia; 12 Upper Gastrointestinal Surgery Unit, Royal Adelaide Hospital, Adelaide, Australia; 13 Upper Gastrointestinal Surgery Unit, Queen Elizabeth Hospital, Woodville, Australia; 14 Discipline of Surgery, The University of Adelaide, Adelaide, Australia; 15 Upper Gastrointestinal Surgery Unit, Northern Health, Epping, Victoria, Australia; 16 Upper Gastrointestinal Surgery Unit, Melbourne Health, Parkville, Victoria, Australia; 17 Upper Gastrointestinal Surgery Unit, Monash Health, Clayton, Victoria, Australia; 18 Upper Gastrointestinal Surgery Unit, Western Health, Footscray, Victoria, Australia; 19 Business Information Unit, Austin Health, Heidelberg, Victoria, Australia; Acibadem Maslak Hospital: Acibadem Maslak Hastanesi, TÜRKIYE

## Abstract

**Introduction:**

Early detection of peritoneal disease, especially micro-metastases, in patients with gastroesophageal adenocarcinoma is critical as it alters therapeutic intent, providing a vital opportunity to personalise treatment. However, our ability to accurately stage the peritoneum is inadequate. Tumour-derived DNA in peritoneal lavage fluid (ptDNA) has been suggested to be more sensitive than current methods to stage the peritoneum. Accordingly, this study will determine whether ptDNA is a biomarker of peritoneal micro-metastasis and evaluate its prognostic value in patients with gastroesophageal adenocarcinoma undergoing curative-intent treatment.

**Methods and analysis:**

This will be an Australian multi-centre prospective observational cohort study enrolling patients undergoing routine staging laparoscopy and subsequent curative-intent treatment (either upfront surgery or perioperative chemo-/radiotherapy and surgery) for gastric and gastroesophageal junction adenocarcinoma. Tumour biopsies, blood and peritoneal lavage fluid will be collected at the time of staging laparoscopy for all patients. A subset of patients will have blood and peritoneal fluid collected at the time of surgical resection, and blood collected at the first post-operative clinic. These biospecimens will undergo genomic and methylomic analysis to detect tumour DNA. ptDNA status will be correlated to disease free survival, peritoneal-specific event free survival, overall survival, sites of treatment failure, histopathological features, and peritoneal lavage cytology status.

**Registration:**

This study is registered with the Australian New Zealand Clinical Trials Registry (ACTRN12624000451505p).

## Introduction

Accurate staging of gastroesophageal cancer is crucial for determining prognosis, treatment intent and approach. Staging typically involves endoscopy, PET/CT imaging and peritoneal lavage cytology. Peritoneal lavage cytology involves irrigating the peritoneal cavity with saline and retrieving this fluid to assess for the presence of cancer cells. Patients without demonstrable distant organ or peritoneal disease are treated with chemotherapy and radical surgery in the belief that aggressive and potentially morbid treatment will deliver a cure. However, many of these patients have locally advanced disease which carries a high risk for trans-coelomic spread into the peritoneum. Unfortunately, the presence of peritoneal metastases is currently considered incurable [1]. Therefore, early and accurate detection of peritoneal disease, especially micro-metastases, is critical as it alters therapeutic intent, providing a vital opportunity to personalise treatment for patients.

Currently, conventional staging methods are unreliable in identifying peritoneal disease. For example, CT/PET imaging is unable to detect peritoneal disease unless advanced features (e.g., nodules or ascites) are present [2]. Despite the pervasive use of peritoneal lavage cytology, its sensitivity for detecting micro-metastases ranges from 10–80%, with 20–40% of tests returning an indeterminant result [3]. Moreover, newer technologies such as testing circulating tumour DNA in blood may not accurately stage the peritoneum [4,5]. As a result, 50% of patients who undergo aggressive treatment with curative-intent, develop early recurrence within 11 months after radical surgery [6,7], and over 50% of these patients recur in the peritoneum as their first site [8]. This suggests that peritoneal micro-metastases were present and missed at the time of staging, and patients have consequently undergone highly morbid treatment for little benefit. Therefore, a sensitive assay to accurately stage the peritoneum is urgently needed to tailor treatments for patients with gastroesophageal cancer, and thus improve survival and quality-of-life outcomes.

Peritoneal tumour DNA (ptDNA) is tumour-derived DNA detectable in either the cellular or cell-free fractions of peritoneal fluid [9]. The presence of ptDNA may indicate the existence of cancer cells within the peritoneum despite a negative or indeterminant CT/PET or peritoneal lavage cytology result [10]. Accordingly, ptDNA identified in peritoneal lavage fluid may be more sensitive than cytology in staging the peritoneum. As proof-of-concept, we performed a systematic review to examine the potential clinical utility of ptDNA in gastroesophageal cancer [10–15]. We found that 1) ptDNA is detectable in peritoneal lavage fluid, 2) ptDNA may be more sensitive than peritoneal lavage cytology in detecting peritoneal metastases, and 3) ptDNA positivity can predict patient survival [16].

Whilst these findings are promising, they also have significant limitations. 1) They were all single institution Asian studies where disease biology and treatment approaches are different to Western countries [17]. 2) They all adopted a tumour agnostic approach, which can lead to a higher false-positive ptDNA detection rate. This is problematic as treatment-intent may be contingent on the specificity of this assay. 3) These studies captured patients across all stages of gastric cancer including those with peritoneal carcinomatosis, thus inflating the true sensitivity of their assay. 4) Most studies examined only one gene, which potentially limits the sensitivity of their assay. 5) Most studies evaluated DNA methylomic analysis of peritoneal fluid with no studies comparing the accuracy of methylomic versus genomic techniques to identify ptDNA. 6) No study has evaluated the accuracy of combining genomic and methylomic approaches to detect ptDNA and predict clinical outcomes. 7) All studies have focussed on gastric cancer, with none examining the role of ptDNA in gastroesophageal junction cancer despite its rapidly rising incidence throughout the world [18]. Addressing these key shortcomings are crucial to translating ptDNA tests into clinical use.

Accordingly, in this study we will use genomic and methylomic techniques [19–21], through tumour informed and agnostic approaches, to analyse ptDNA in peritoneal lavage fluid from patients who are undergoing curative-intent treatment in high-volume Australian centres.

### Hypotheses

We hypothesize that ptDNA is a biomarker of peritoneal micro-metastasis and prognosticates survival in patients with gastroesophageal adenocarcinoma undergoing curative-intent treatment.

### Study aims

1. To determine whether pre-treatment ptDNA, detected using genomic and methylomic approaches, predicts DFS in gastroesophageal cancer patients.
2. To compare the performance of ptDNA versus conventional staging methods (peritoneal lavage cytology, PET/CT) to predict histopathological features of the tumour, patterns of disease recurrence, and survival.
3. To compare ptDNA detection rates before (at time of peritoneal lavage cytology) and after (at time of surgical resection) neoadjuvant therapy.
4. To compare ptDNA versus circulating tumour DNA to predict sites and patterns of disease recurrence.
5. To compare the cost-effectiveness of genomic versus methylomic approaches to detect ptDNA to inform translation into clinical practice.

## Methods

### Study design

This is a prospective observational cohort study conducted across 9 oesophago-gastric cancer centres in Australia (S1 Table in S1 Supporting material). All consenting patients with a histologically confirmed gastroesophageal junction or gastric adenocarcinoma, who are ≥18 years-of-age and undergoing staging laparoscopy for peritoneal lavage cytology are eligible to participate in this study. Exclusion criteria include patients with 1) a primary oesophageal cancer, 2) radiological evidence of visceral, non-regional nodal, and peritoneal metastases, 3) indications requiring emergency surgery, 4) an European Cooperative Oncology Group performance status ≥3, and 5) patients who receive palliative-intent treatment.

### Study overview

**Fig 1:**
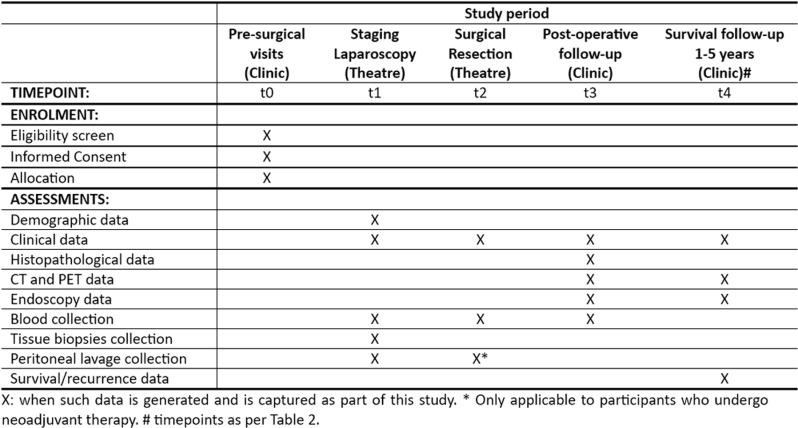
SPIRIT Schedule of enrolment and assessments.

**Fig 2:**
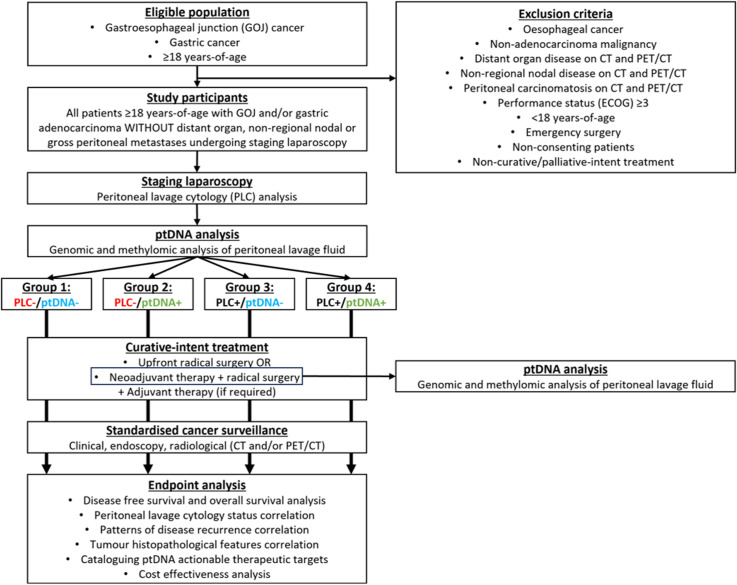
Schema of clinical trial.

Tumour and normal (stomach and oesophagus) tissue biopsies, blood, and peritoneal lavage fluid will be collected during pre-treatment staging for all participants in this study (Fig 1). For those who undergo neoadjuvant chemo/radiotherapy, tumour tissue, blood and peritoneal lavage fluid will also be collected at time of surgery. Additionally, blood will be collected in outpatient clinics 4–6 weeks after surgical resection. These samples will be centralised to one laboratory and undergo DNA extraction, with portions reserved for methylomic and genomic sequencing to identify the presence or absence of ptDNA (in peritoneal fluid) and circulating tumour DNA (in plasma). Moreover, clinical data will be entered into an online REDCap database by collaborators from each hospital. Each patient’s ptDNA and circulating tumour DNA status will be correlated with survival, clinical, radiological, and pathological endpoints (Fig 2). Participant recruitment will occur over 2 years (starting 1^st^ August 2024) with clinical follow-up for 5 years after the last enrolled patient. This study has been approved by the Peter MacCallum Cancer Centre Human Research Ethics Committee (HREC/106535/PMCC) and registered with the Australian New Zealand Clinical Trials Registry (ACTRN12624000451505p). This study protocol has been written according to the STROBE guidelines.

### Study endpoints and definitions

The primary endpoint for this study is 2-year disease free survival (DFS). Secondary endpoints include overall survival (OS), peritoneal-specific event free survival, sites of treatment failure, tumour histopathological features, sensitivity and specificity of ptDNA and circulating tumour DNA against peritoneal lavage cytology status, additional detection rate of ptDNA, and cost metrics. DFS will be calculated from the date of surgery to the date of disease recurrence at any site, as determined by clinical, endoscopic, and/or radiological examination. Peritoneal-specific event free survival will be calculated from the date of peritoneal lavage to the date of peritoneal recurrence as determined by radiological and/or laparoscopic investigations. OS will be calculated from the date of surgery to the date of death from any cause. Those alive at study termination will be censored at the time of their last assessment. The additional detection rate of ptDNA is defined as the ratio of ptDNA-positivity/cytology-negativity [16]. Baseline comorbidities will be quantified using the Charlson Comorbidity Index [22]. Clinicians’ assessment of each patient’s performance status and overall surgical fitness will be classified using the Eastern Cooperative Oncology Group scale [23] and the American Society of Anesthesiologist score [24], respectively. Postoperative complications will be defined according to the European Perioperative Clinical Outcome definitions [25], with specific surgical complications defined by the Esophagectomy Complication Consensus Guidelines [26]. The severity of each adverse event will be classified using the Clavien-Dindo system with a major complication defined as grade ≥3 [27]. Gastroesophageal junction tumours will be categorized according to Siewert’s classification [28]. Cancer staging including T, N and M stage for CT/PET scans and histopathology will be according to the 8^th^ edition AJCC Cancer Staging Manual [29]. We have decided to use DFS rather than peritoneal-specific event free survival as our primary endpoint because surveillance endoscopy and cross-sectional imaging are more sensitive at detecting non-peritoneal recurrences than peritoneal metastases.

### Recruitment procedure and consent process

Potential study participants will be identified through hospital outpatient clinics and approached by the treating clinician and/or local investigators. A verbal description of the study together with the patient information consent form (**Supporting material 2–4**) will be provided. The purpose, requirements and risks of the study will be explained in a clear manner. The clinician/investigator will allow time to address any questions from the patient and will also emphasize that the standard of care will not be altered in any way, regardless of the patient’s consent. Professional interpreters may be used where appropriate to translate the discussion. Any patient who is not able to give informed consent will be excluded. All procedures will be conducted in accordance with the Declaration of Helsinki. Identification and recruitment of patients, as well as data collection will be performed by health professionals within the treating team.

### Clinical data collection

Clinical data will be collated through an online REDCap database. This will include patient demographics, co-morbidities and performance status, cancer staging (CT, PET/CT, endoscopy and laparoscopy data), neoadjuvant treatment, surgery and perioperative outcomes, tumour histopathology, adjuvant treatment, survival endpoints, sites of treatment failure, management of recurrent disease, and cost data associated with clinical episodes of care. Data entry will be performed by designated clinicians already employed at each centre. Multiple quality assurance measures will be applied to minimise inter-observer discrepancies in data entry. These include the use of a standardized data collection tool (developed, tested and validated by the Peter MacCallum Data Systems, Research Computing Facility), training sessions for data collectors, in-program prompting, and real-time data entry support. Data cleaning will be conducted independently by two investigators. A random audit of 10% of data fields, by cross-referencing with patient medical records will be undertaken to quantify the accuracy of entered data from all sites. This snapshot audit provided through REDCap analysis tools has been widely validated across multiple datasets demonstrating high levels of case ascertainment (typically 90–95%) and data accuracy (96–98%) [30,31]. This audit will be performed by a collaborator at each centre who were not involved in the primary data collection.

### Standardising peritoneal lavage collection

At staging laparoscopy, a total of 500 ml of 0.9% saline wash will be applied to all four quadrants of the peritoneum and collected. From this half will be submitted to anatomical pathology for routine processing and cytological analysis, and the remainder will be used for ptDNA detection. For patients who have undergone neoadjuvant therapy, a further peritoneal lavage will be performed at surgical resection. Peritoneal lavage will be performed immediately after achieving peritoneal access. In total, 500 ml of 0.9% saline wash will be applied to all four quadrants of the peritoneum and collected. From this 200 ml will be used for ptDNA detection and the remainder will be discarded.

### Biospecimen collection

Peritoneal lavage, venipuncture, endoscopy and biopsies will be performed by the clinical treating teams at each site. The timepoints and type of biospecimens to be collected is summarized in Table 1.

**Table 1:** Types and timing of biospecimen collection.

Biospecimens	At staging laparoscopy	At surgical resection	At post-surgery clinic
Peripheral blood	20–30 ml Prior to skin incision	20–30 ml Prior to skin incision	20–30 ml
Primary tumour tissue	8–12 total endoscopic biopsies 4–6 in normal saline 4–6 in formalin	–	–
Normal stomach tissue	4–6 endoscopic biopsies in normal saline	–	–
Normal oesophageal tissue	4–6 endoscopic biopsies In normal saline	–	–
Peritoneal fluid	200 ml of 500 ml 0.9% saline 4 quadrants peritoneal lavage	200 ml of 500ml 0.9% saline 4 quadrant peritoneal lavage at start of surgery^*^	–

* For participants who have undergone neoadjuvant treatment

### Follow-up and survival endpoint detection

Clinical follow-up will take place for 5 years after the last enrolled participant. All participants will undergo outpatient, endoscopic and imaging assessment as per their local hospital guidelines. This is typically as per Table 2. For participants who fail to attend follow-up, local clinicians and PIs at each site will attempt to contact these participants, as per standard-of-care, via telephone or videoconferencing to attain clinical follow-up and outcome data.

**Table 2:** Follow up schedule.

Assessments and procedures	0–1 year	1–3 year	3–5 year
Clinical	3 monthly reviews	6 monthly reviews	Annual reviews
Imaging CT and/or PET	At 1 year	Annually	Annually
Endoscopy	At 1 year	At 2^nd^ and 3^rd^ year	–

### Genomic and methylomic analysis

DNA from peritoneal lavage fluid, plasma, and primary cancer tissue will be extracted. These samples will be analysed using: 1) Tumour-informed genomic analysis, and 2) Tumour-agnostic methylomic analysis.

For tumour-informed genomic analysis: Tumour specific mutations will be identified via whole genome sequencing of the primary tumour DNA and blood derived buffy coat DNA to identify up to 96 tumour specific variants individualised to each patient. Targeted, amplicon based sequencing (specific to each patient’s tumour) will be performed on DNA derived from peritoneal lavage fluid and plasma, resulting in a binary output of positive or negative for the presence of ptDNA and ctDNA, respectively [21,32,33].

For tumour-agnostic methylomic analysis: Characteristic tumour hypermethylation markers, consisting of the top differentially methylated regions between gastroesophageal cancer versus normal oesophageal, stomach and peritoneal tissues will be quantified using a targeted DNA methylation next generation sequencing panel. DNA derived from peritoneal lavage fluid will be sequenced to identify DNA methylation status from these markers, resulting in a final binary output of positive or negative for the presence of ptDNA [34,35].

### Sample size calculation

This study will have 4 observational cohorts (Fig 2): Group 1: Peritoneal lavage cytology-negative and ptDNA-negative, Group 2: Peritoneal lavage cytology-negative and ptDNA-positive, Group 3: Peritoneal lavage cytology-positive and ptDNA-negative, and Group 4: Peritoneal lavage cytology-positive and ptDNA-positive. However, as the primary objective of this study is to evaluate the clinical utility of ptDNA as a biomarker to predict the primary endpoint (2-year DFS) in patients with clinically occult micro-metastatic disease in the peritoneum (using current ‘best practice’ staging methods), we have powered this study to compare groups 1 and 2, as these are the patients with peritoneal lavage cytology-negative disease. Accordingly, we deemed a 30% absolute DFS difference at 2 years post-surgery as clinically significant between groups 1 and 2. This is based on our hypothesis that patients who are ptDNA-positive should have cytology-positive disease, even if this was not detected. Based published data, the conservative estimate of 2-year DFS in patients who are cytology-positive versus cytology-negative is 40% versus 70%, respectively [3,10]. Using our pilot data and our systematic review [16], we anticipate approximately 1 in 8 patients who is cytology-negative will be ptDNA-positive, this means an enrolment ratio of 7–1 in groups 1 and 2, respectively. To detect a 30% absolute difference in 2-year DFS between these two groups, we would need to enrol 200 patients with 175 patients in group 1 (ptDNA-negative arm) and 25 patients in group 2 (ptDNA-positive arm) to reach 80% statistical power at an alpha <0.05.

### Data analysis plan

Interim analyses: We will perform an interim analysis at 1 and 2 years after commencement of recruitment to ensure that the enrolment ratio between groups 1 (cytology-negative/ptDNA-negative) and 2 (cytology-negative/ptDNA-positive) is on-track (Fig 2). This will be determined by analysing the proportion of patients who are cytology-negative and ptDNA-positive. If the ratio of ptDNA-positive/cytology-negative is ≥10%, then recruitment of patients will continue to the target of 200. If this ratio is <10%, we will adjust our recruitment strategy accordingly. Additionally, we will perform an interim analysis at 3 years after commencement of recruitment (≥1-year DFS data) to compare DFS between groups.

Final analyses: The clinical utility of ptDNA (ptDNA-positive vs ptDNA-negative), as determined by genomic and methylomic output, will be evaluated using the following metrics: sensitivity, specificity, positive-predictive value, negative-predictive value, additional detection rate, receiver operator characteristics, Kaplan Meier and Cox regression, against the primary and secondary endpoints. Co-variates in these analyses will be accounted for using hierarchical multi-variate logistic regression algorithms.

Exploratory analyses will be performed to:

1) Compare ptDNA detection rate before (at time of staging laparoscopy) and after (at time of surgical resection) neoadjuvant therapy.
2) Compare ptDNA versus circulating tumour DNA to predict sites and patterns of disease recurrence.
3) Compare the cost-effectiveness of genomic versus methylomic approaches to detect ptDNA to inform translation into clinical practice. Using data collected including admission and discharge dates, length of stay, clinical services provided to each episode of care (e.g., CT, PET, laparoscopy, chemotherapy, radiotherapy, surgery etc.), outpatient follow-up, treating clinical units, and estimated unit costs based on diagnosis-related group, we will calculate the actual cost associated with each patient’s care along their cancer treatment journey. Then at a population level, this will be compared to a hypothetical projected cost based on whether ptDNA status changes clinical management, and whether this cost difference offsets the cost of a ptDNA assay.

These analyses will be performed using chi-square tests, Pearson correlations and other quantitative methodologies.

### Administrative aspects

#### Handling of withdrawals and replacements.

Patients may choose to withdraw from the study at any time at their own volition. Should a patient withdraw consent from further participation in this study, they will undergo routine clinical care without any additional biospecimen collection and all clinical data collection relating to the patient will cease. Wherever possible, all collected and analysed data will be electronically and/or manually deleted from our study records. All collected tissues and fluid will be disposed of appropriately. This will not impact on our ability to use samples and data obtained from other participants and therefore will not have any overall adverse impact on our project. We plan to replace all withdrawn participants through additional recruitment to ensure that this study meets the required sample size.

#### Data security and handling.

All data will be de-identified before entry into REDCap database. This database will be hosted at the Peter MacCallum Cancer Centre and governed by the hospital’s information technology and security processes. This includes appropriate best practices such as network firewalls, system and security monitoring and two-factor authentication. REDCap also implements authentication to validate the identity of users that log in to the system. REDCap maintains an audit trail that logs user activity, including contextual information (e.g., the project or record being edited). The logging record can be viewed by users who have appropriate privileges. REDCap access privileges will be managed and maintained by the coordinating principal investigators and study coordinator/project manager alongside Peter MacCallum Cancer Centre REDCap managers. The coordinating principal investigators, database manager, and statistician will have access to the entire database for database monitoring and analytical purposes. Principal investigators at each site can only view their own hospital’s data. Patient identifiers will be replaced with a unique study number. The site-specific master list of names and matching codes will be stored on password protected network at each participating site, with access to them only by staff directly involved with the project as determined by site principal investigator. This will enable re-identification should this situation arise. These site-specific master lists will be destroyed once the project is closed. No identifiable information will be shared with collaborators outside the study investigators unless otherwise specified in an agreement or approved protocol. Data will be stored for at least 15 years after the completion of research activity.

Additionally, the following steps will also be undertaken to maintain the confidentiality of patients and their clinical data. Firstly, tissue and clinical data will be coded with a unique study number. The linker between the study number and patient identifier will be stored locally at each site. This will allow for the sample to be linked back to the participant’s medical records if required. Secondly, all data generated from this study will remain confidential and no published work will contain patient identifiers. Thirdly, any publications or presentations that arise from this project will be presented as general cohort information with numbers and statistics. No individual data will be published or shared to ensure that identification of individual patients is not possible. Finally, all study-related personnel are bound by professional standards of patient information confidentiality and will work to always protect patient confidentiality.

#### Publication and curation.

Study outcomes will be largely disseminated through peer-reviewed scientific literature, conference presentations and seminars. In any publication and/or presentation, information will be provided in such a way that individual participants cannot be identified, except with their permission. There is no formal plan to return results of this study to participants who provide tissue, blood samples or peritoneal fluid, except in the rare case of incidental findings that may have an impact on the health of the participant or their family, and the participant has consented to being contacted.

#### Patient and public involvement.

Two patient advocates, who are themselves survivors of gastroesophageal cancer, were involved in the design, grant application, protocol review, and steering committee for this study. Our research question was informed by their lived experiences. Additionally, as part of the protocol development and ethics application process, our two patient advocates were intimately involved in assessing the potential burden of obtaining additional biospecimens for research from participants in this study. Our patient advocates are invitees to our quarterly project steering committee meetings. They will also be given opportunities to disseminate the findings from this study to the wider patient community through our cancer survivorship networks and community support groups.

#### Study timeline.

The anticipated timeline for this study is detail below in Table 3.

**Table 3:** Study timeline.

Milestone	Anticipated date
Start of participant recruitment:	August 2024
End of participant recruitment:	December 2025
Completion of study visits:	December 2030
Interim analysis:	February 2025, 2026, 2027
End of Study:	December 2030
Final study report:	Feb 2031
Planned publication/presentation:	May 2031

## Discussion

Due to the high propensity for trans-coelomic spread of gastroesophageal cancer to the peritoneum [36], accurate staging of the peritoneal cavity is critical for clinical decision-making. Early detection of peritoneal disease, especially micro-metastases, may alter treatment-intent and approach. Currently, our ability to accurately stage the peritoneum is inadequate. This means that at least 20–30% of patients are under-staged, and therefore inappropriately offered aggressive, potentially morbid and futile treatments [16]. Therefore, a sensitive assay to accurately stage the peritoneum may enable the appropriate triaging of therapies to avoid over- or under-treating patients with gastroesophageal cancer.

This study will address critical limitations in the literature. First, to our knowledge, this will be the first multi-centre study of ptDNA in gastroesophageal cancer from a Western population. Second, this study will investigate the potential complementary role of both methylomic and genomic approaches in detecting ptDNA in overcoming issues of false-positivity and false-negativity. Third, as we have excluded patients with stage 4 disease, peritoneal carcinomatosis, and those undergoing non-curative intent treatment, we will be assessing ptDNA as a biomarker of occult peritoneal disease thus reflecting the true utility of this assay. Fourth, our approach will utilise a proven approach to detecting low frequency DNA variants and a validated panel of differentially methylated genes to identify ptDNA [21,32,33,37], thus maximising the sensitivity of this assay. Fifth, this study will compare the accuracy and cost-effectiveness of methylomic versus genomic approaches to inform future clinical trials and translation into clinical practice. Finally, this study will enrol patients with gastric and gastroesophageal junction adenocarcinomas, thus broadening the generalisability of this ptDNA assay.

The low concentration of cell-free DNA in plasma and peritoneal fluid, particularly in early-stage disease, is an important consideration and may impact on the sensitivity of our assays. To address this, we have optimised our DNA extraction methodology to maximise DNA yield. We will utilise PCR-based techniques to amplify low levels of cell-free DNA. Moreover, our laboratory has a proven track record of detecting low frequency DNA variants in bodily fluids using genomic and methylomic platforms [21,32,33,37].

If successful, our ptDNA assay may be incorporated into disease prognostication and patient counselling. Moreover, our findings will inform future trials to evaluate whether ptDNA status can be used to guide escalation or de-escalation of treatment, as well as using ptDNA as a companion biomarker for selecting patients into peritoneal-directed therapeutic trials.

## Supporting information

S1 Supporting material S1 Table. Participating hospitals. (DOCX)

S2 Supporting materials S2 Patient Information and Consent Form. (DOCX)

S3 Supporting materials S3 Study protocol. (DOCX)

S4 Supporting materials S4 SPIRIT checklist. (DOCX)
