# Supplementary material for: Multi-omics evaluation of peritoneal fluid in gastroesophageal cancer (OMEGCA): protocol for a prospective multicentre cohort study to detect occult peritoneal metastases in patients undergoing curative-intent treatment
Source: PLoS One. 2025 Apr 16;20(4):e0318615. doi: 10.1371/journal.pone.0318615 (PMC12002517; doi:10.1371/journal.pone.0318615)
Supplement: S1 Supporting material — (DOCX) [file pone.0318615.s001.docx]

**Supporting materials**

**Table S1.** Participating hospitals

| **Hospital name** | **State** | **Country** |
| --- | --- | --- |
| Peter MacCallum Cancer Centre | Victoria | Australia |
| Austin Health | Victoria | Australia |
| Northern Health | Victoria | Australia |
| Monash Health | Victoria | Australia |
| Melbourne Health | Victoria | Australia |
| Western Health | Victoria | Australia |
| Flinders Medical Centre | South Australia | Australia |
| Royal Adelaide Hospital | South Australia | Australia |
| Queen Elizabeth Hospital | South Australia | Australia |
