# Supplementary material for: Multi-omics evaluation of peritoneal fluid in gastroesophageal cancer (OMEGCA): protocol for a prospective multicentre cohort study to detect occult peritoneal metastases in patients undergoing curative-intent treatment
Source: PLoS One. 2025 Apr 16;20(4):e0318615. doi: 10.1371/journal.pone.0318615 (PMC12002517; doi:10.1371/journal.pone.0318615)
Supplement: S2 Supporting materials — (DOCX) [file pone.0318615.s002.docx]

*Insert Header with institution’s name or institution’s letterhead*

**Participant Information Sheet/Consent Form**

*[Insert site name]*

| **Title** | *Multi-Omics Evaluation of Peritoneal Fluid in Gastroesophageal Cancer (OMEGCA): A prospective trial to develop a sensitive assay to detect clinically occult peritoneal metastases* |
| --- | --- |
| **Coordinating Principal Investigator/ Principal Investigator** | *Dr. David Liu*  *A/Prof. Nicholas Clemons* |
| **Associate Investigator(s)**  *(if required by institution)* | *Prof. Niall Tebbutt, Prof. Jeanne Tie, Dr. Stephen Wong, Prof. David Watson, Dr. Markus Trochsler, Dr. Margaret Lee, Dr. Darren Wong, A/Prof, Ronald Ma, Dr. Zexi Allan, Dr. Krinal Mori, Dr. Nicole Winter, Dr. Sarah Martin, Dr. Geraldine Ooi, Dr. Yahya Al-Habbal, and Ms. Katheryn Hall* |
| **Location** *(where CPI/PI will recruit)* | *Peter MacCallum Cancer Centre, VIC*  *Austin Health, VIC*  *Northern Health, VIC*  *St Vincent’s Health, VIC*  *Melbourne Health, VIC*  *Western Health, VIC*  *Eastern Health, VIC*  *Monash Health, VIC*  *Flinders Medical Centre, SA*  *Royal Adelaide Hospital, SA*  *Queen Elizabeth Hospital, SA* |

**Part 1 What does my participation involve?**

**1 Introduction**

You are invited to take part in this research study which has the potential to impact the future treatment and care of patients with oesophageal and stomach cancer. This is because you are having an endoscopy, laparoscopy and/or surgery for your oesophageal or stomach cancer. This study is aiming to develop an accurate molecular test to detect microscopic spread of cancer cells into the abdominal cavity. We believe this information will facilitate patient counselling, inform clinical decision-making, and help personalise cancer treatment to maximise benefit and reduce harm.

This Participant Information Sheet/Consent Form tells you about the research study. It explains the tests and research involved. Knowing what is involved will help you decide if you want to take part in the research.

We invite you to read this information carefully. Ask questions about anything that you don’t understand or want to know more about. Before deciding whether or not to take part, you might want to talk about it with a relative, friend or your local doctor.

Participation in this research is voluntary. If you don’t wish to take part, you don’t have to. You will receive the best possible care whether or not you take part.

If you decide you want to take part in the research study, you will be asked to sign the consent section. By signing it you are telling us that you:

• Understand what you have read

• Consent to take part in the research study

• Consent to the tests and research that are described

• Consent to the use of your personal and health information as described.

You will be given a copy of this Participant Information Sheet/Consent Form to keep.

**2 What is the purpose of this research?**

One of the cornerstones of treating patients with stomach and oesophageal cancer is cancer staging. Cancer staging is assessing the extent of disease spread in the body when people first present with their diagnosis. Determining the cancer stage allows doctors to predict the likely future prognosis of this disease, and most importantly, enables patients and their doctors to select the correct treatment-intent (curable or non-curable), and treatment approach (chemotherapy, radiotherapy, surgery or combinations of, or non-surgical approaches) to best manage their cancer.

Stomach and oesophageal cancers have a high likelihood of spreading to the abdominal cavity (peritoneum). The presence of peritoneal disease is currently considered difficult to cure. Unfortunately, despite our best staging tests (CT and PET scans, and peritoneal washings to detect cancer cells), our ability to accurately stage the peritoneum is inadequate. This means that many patients might be offered treatments that come with significant side effects. Therefore, an accurate test to stage the peritoneum is urgently needed to personalise treatment and avoid over-treating patients with stomach and oesophageal cancer.

Oesophageal and stomach cancers release genetic material known as DNA into their surroundings like blood and the peritoneum. The presence of DNA may suggest that cancer cells have spread into that area. ptDNA is cancer-derived DNA detectable in peritoneal washings. We propose that ptDNA detection may be more accurate than current methods to examine the peritoneum. In our laboratory, we have developed the ability to detect ptDNA.

In this study, we would like to test your peritoneal washings for ptDNA. We will compare the performance of two different methods to detect ptDNA and determine whether ptDNA status correlates with clinical outcomes.

If successful, this study will produce a novel and accurate molecular test to detect microscopic peritoneal cancer deposits. This information will improve disease staging, facilitate patient counselling, inform clinical decision-making, and personalise cancer treatment to maximise benefit and reduce harm.

This research has been funded, in part, by grants from the Australian Gastro-Intestinal Trials Group and the Victorian Cancer Agency.

**3 What does participation in this research involve?**

In this project we will study tissue, blood samples and peritoneal fluid from participants with oesophageal and stomach cancer.

You have been invited to take part in this study because you are having an endoscopy, laparoscopy, and/or surgery in which your doctor will look at the lining of the inside or outside of your oesophagus/stomach and take tissue biopsies (small samples) as well as peritoneal washings for diagnostic purposes.

If you decide you would like to take part in this study, we will:

- **At the endoscopy/staging laparoscopy stage**: collect some *extra* biopsies, a sample of blood (~20-30 ml or about 2 tablespoons) and *extra* peritoneal wash fluid whilst you undergo your procedure.
- **At the surgery stage**: collect a sample of blood (~20-30 ml), and peritoneal wash fluid whilst you undergo your procedure.
- **Following surgery/outpatient review stage**: collect a sample of blood (~20-30 ml)
- **Clinical follow-up after all treatment is complete**: collect health information including staging, tumour features, treatment details and survival outcomes on an ongoing basis.

**Tissue biopsies**: The biopsies are each about the size of a quarter of a grain of rice. Eight to twelve extra biopsies will be taken from the tumour, and four to six extra biopsies will be taken from normal appearing oesophagus and stomach. These research biopsies will only be taken after the diagnostic biopsies have been collected and at the discretion of your doctor.

**Blood collection:** At the time of staging laparoscopy and surgery, as part of normal clinical care, a cannular (plastic tube) will be inserted by your anaesthetist into your blood vessels in your arm to give you medications and draw blood samples for your treatment. As part of this 20-30 ml of blood will be drawn for research purposes. During outpatient review, a small needle will be inserted into your arm veins to draw 20-30 ml of blood for research purposes.

**Peritoneal fluid collection:** Typically, your surgeon will wash the abdominal cavity with several hundred mls of saline (salt water) fluid and collect this fluid to look for cancer cells. This fluid is discarded afterwards. We would keep 150-250 ml of this wash fluid for research purposes.

Often some of the material used for the diagnostic tests is left over, and we would also like to access this remaining tissue (called a tissue block) for research. The diagnostic blocks would only be accessed once the pathologist has completed all the necessary diagnostic tests and no longer needs the blocks.

We would also like your permission for the doctors conducting the study to access your relevant medical and pathology records (including preserved tissue blocks from previous diagnostic testing, if appropriate) to correlate with the results of the tissue, blood and peritoneal fluid analysis.

There are no costs associated with participating in this research study, nor will you be paid. There is a chance that information derived from the samples that you are donating under this study may, in the future, have some commercial value, for example if they lead to the development of a commercial product. You will not be compensated for your participation in the study or for any future value that the sample you have given may be found to have.

**4 What do I have to do?**

If you agree to participate in this research study, you will be asked to sign the consent form at the end of this document consenting to the collection of the samples (and associated data) outlined above in section 3.

The provision of these samples and your consent for their use as outlined in this document is the only requirement for your participation in this study. Your direct involvement will end once the samples have been collected.

We may contact you again in the future to ask you to provide further samples or to ask if you would like to take part in a follow-up study but you will be under no obligation to do so.

**5 Do I have to take part in this research study?**

Participation in any research study is voluntary. If you do not wish to take part, you do not have to. If you decide to take part and later change your mind, you are free to withdraw from the study at any stage.

Your decision whether to take part or not to take part, or to take part and then withdraw, will not affect your routine treatment, your relationship with those treating you or your relationship with your hospital.

**6 Will I find out the results of the research using my tissue or blood?**

You will not be given individual results from this research. This is because research can take a long time and must use tissue samples from many people before the results are known. Also, as this kind of research by its very nature is experimental, any new discoveries may take considerable time before they can be translated into clinically relevant results. However, upon request to the Principal Investigator, we will provide you with a summary of the study results, keeping in mind that it could be several years before results are known. Furthermore, if research findings are made that may have significant implications for you or your family, you will be given the chance to learn more about this information if you wish (see section 8).

**7 What are the possible benefits of taking part?**

There will be no clear benefit to you from your participation in this research. However, the results of this study may help improve the treatment and care of people with similar problems of the oesophagus and stomach in the future.

**8 What are the possible risks and disadvantages of taking part?**

While this research does not involve any additional procedures, there are minor risks involved.

**Tissue biopsy***:* Taking *extra* biopsies has minimal risk of harm as you are already having an endoscopy/laparoscopy and the biopsies themselves are very small. There is a small risk of bleeding with any biopsy collection; however there is no significant additional risk from taking extra biopsies for research purposes. No additional endoscopic/laparoscopic procedures will be performed other than those being undertaken for diagnostic purposes. However, taking biopsies for research means your procedure will take a few minutes longer.

**Blood collection***:* Having a blood sample taken may cause some discomfort, bruising, minor infection or bleeding. If this happens, it can be easily treated. In most cases, taking blood for this study will not involve any additional discomfort or risk since wherever possible this will be collected through the cannula which is routinely inserted into a vein in your arm prior to your endoscopy, laparoscopy or surgery.

**Peritoneal fluid collection**: Collecting this fluid has no additional risk of harm as you are already having this fluid collected as part of a staging laparoscopy. During surgery, saline fluid is commonly used to wash the abdominal cavity and then removed for the purpose of cleaning the operative field.

**Genetic Research***:* Genetic research involves the study of genetic material (DNA), which is shared with your blood relatives. Genetic research raises many important issues. It is unlikely that these issues will arise, but you should think about them carefully. There is a very small possibility that future research using your samples may result in new genetic information about your specific disease or your risk of getting other conditions.

From here on, a patient’s “**information”** refers to personal, health and genetic information.

If research findings are made that may have significant implications for you or your family, the researcher will submit a full report to the Peter MacCallum Human Research Ethics Committee who will then consider whether you or your family might benefit from the chance to learn more about this information. It is your choice whether or not you wish to know of any important results and we will ask you if you want us to tell you if we do find any information that has significant implications for you or your family. We will also ask you if you want the information to be given to a member of your family if we cannot contact you. We will not give any information about you to members of your family without your permission.

If we find some important information, and you have declared that you do wish to know of any significant results, we will do our best to contact you to ask if you still want to find out more about these findings. At that time, you would then be given genetic counselling about the implications of receiving genetic information and, if you then decided that you wished to obtain your individual results, you would be asked to give a second blood sample so that the research results could be confirmed in an accredited testing laboratory. We will not be able to give you results directly but, with your permission, we will pass information onto a clinical testing laboratory so that they can easily repeat the test under the appropriate testing conditions.

Currently in Australia, genetic testing does not affect your ability to obtain private health insurance (although there may be a waiting period for pre-existing conditions). It is, however, possible that in the future the fact that you have taken part in a study involving genetics may affect you or your family if you want to take out a new life or income protection insurance policy or increase an existing life or income protection insurance policy. For instance, you may have to disclose whether you have attended a genetic counselling service when you apply for a new life or income protection insurance policy or an increase to an existing life or income protection policy, and this may affect your policy. We will not pass on this information about you to anyone, including your family members, without your written permission unless required to by law.

**9 What will happen to my test samples?**

After collection of tissue biopsies, blood and peritoneal fluid, these samples will be transferred to the Peter MacCallum Cancer Centre for laboratory processing. In a portion of these samples, DNA will be extracted from the cells contained within these samples. The remaining portions will be stored for future analysis and validation tests.

A portion of DNA will be kept at the Peter MacCallum Cancer Centre for further analysis. Some extracted DNA samples, tissues biopsies, blood, and peritoneal fluid samples will be sent to our research collaborators at Johns Hopkins University Hospital, USA and Haystack Oncology^Pty Ltd^ (A specialist DNA analysis company), USA, for further analysis. The results of these analyses will be correlated to the clinical outcomes from the participants enrolled in this study as a group. These correlations will help us determine whether DNA analysis from tissues biopsies, blood and peritoneal fluid will be useful as a diagnostic test.

Your tissue biopsies, blood, peritoneal fluid, DNA, and associated information will be stored securely in a laboratory at the Peter MacCallum Cancer Centre. The samples and DNA sent to our collaborators overseas will also be stored securely in their laboratory. All samples and DNA will be stored in an individually re-identifiable (coded) form to ensure the correct data is collected for you and access will be restricted to approved researchers.

Your tissues may also be provided to other qualified medical researchers from such areas as hospitals, universities, medical research institutes, government affiliated institutions and commercial organisations such as pharmaceutical companies (including overseas institutes and organisations) but only after approval by the Peter MacCallum Human Research Ethics Committee. Any information or material given to researchers will be identified only by a code so it will be impossible for them to identify you in any way.

Your tissues, blood, fluid, DNA, and associated information will NOT be sold. We may charge researchers a fee to recover some of the costs of storing and administering its collection of tissues, but tissues are never sold. If your samples are transferred to another organisation we will not be able to control whether they transfer or sell your samples at some future date, however we will not knowingly transfer your samples to anyone who has expressed intent to sell the samples. Also, although knowledge acquired through medical research may lead to discoveries that are of commercial value to the researcher and their institution, there will be no financial benefit to yourself or your family.

*Optional Future Research*

Any samples not used immediately will be stored for 20 years and may be used for optional future research unrelated to this current research study. Use of your samples in future research is optional and you will be given the opportunity to indicate your preference on the consent page of this document.

Any future unrelated studies that address different aims and research questions, which uses your samples will have to be approved by an appropriately constituted Human Research and Ethics Committee before your information and material can be used. You will not receive any notice of future uses of your information or samples.

If research findings are identified in future research that may have significant implications for you or your family, and you have declared in this consent form that you do wish to know about any significant results, we will following the processes outlined in section 8 above to inform you and/or your family of this information.

**10 What if I withdraw from this research study?**

You may withdraw from the study at any time and this will in no way affect your medical treatment or your relationship with your doctors or with your hospital, now or in the future. Furthermore, you may request for your samples and medical information collected as part of this study to be destroyed and removed from analysis at the time of withdrawing from the study.

Your direct involvement in the research study will end once the samples have been collected. However, if after donating your samples you change your mind and decide that you do not want your samples used for research, let us know that you do not want us to use your samples and any remaining tissues or blood that has not already been used will then be destroyed wherever possible. Occasionally, it may not be possible to destroy material that has already been processed and is part of an established resource.

**Part 2 How is the research study being conducted?**

**11 What will happen to information about me?**

A patient’s “information” refers to personal, health and genetic information.

By signing the consent form you consent to the study doctor and relevant research staff collecting and using information about you for the research study. Any information obtained in connection with this research study that can identify you will remain confidential and securely stored.

Biological samples and data will be coded with an unique alpha-numeric identifier (code). A database linking the unique code with you (for re-identification purposes) will be stored separately on a secure server at the Peter MacCallum Cancer Centre. Only the Principal Investigators will have access to this database. Your information will only be used for the purpose of this research study and it will only be disclosed with your permission, except as required by law. Information collected for this research will be stored for at least 15 years from the end of the study, in accordance with legal requirements, after which time it will be destroyed.

Information about you may be obtained from your health records held at this and other health services for the purpose of this research. By signing the consent form you agree to the research team accessing your health records if they are relevant to your participation in this research study.

Your health records and any information obtained during the research study are subject to inspection for the purpose of verifying the procedures and the data. This review may be carried out by the relevant authorities and authorised representatives of the institution relevant to this Participant Information Sheet, the participating hospital, or as required by law. By signing the Consent Form, you authorise release of, or access to, this confidential information to the relevant research personnel and regulatory authorities as noted above.

It is anticipated that the results of this research will be published and/or presented in a variety of forums. In any publication and/or presentation, information will be provided in such a way that you cannot be identified, except with your permission.

Information about your participation in this research study may be recorded in your health records. In accordance with relevant Australian and/or Victorian privacy and other relevant laws, you have the right to request access to the information collected and stored by the research team about you. You also have the right to request that any information with which you disagree be corrected. Please contact the research team member named at the end of this document if you would like to access your information.

**12 Who has reviewed the research study?**

All research in Australia involving humans is reviewed by an independent group of people called a Human Research Ethics Committee (HREC). The ethical aspects of this research study have been approved by the HREC of the Peter MacCallum Cancer Centre. This study will be carried out according to the *National Statement on Ethical Conduct in Human Research (2007)*. This statement has been developed to protect the interests of people who agree to participate in human research studies.

**13 Further information and who to contact**

The person you may need to contact will depend on the nature of your query.

If you want any further information concerning this study, you can contact the Co-ordinating Principal Investigator, Dr. David Liu at david.liu@petermac.org or A/Prof. Nicholas Clemons at nicholas.clemons@petermac.org or on 03 85595273, or any of the following people:

**Clinical contact person**

| Name | *[Name]* |
| --- | --- |
| Position | *[Position]* |
| Telephone | *[Phone number]* |
| Email | *[Email address]* |

For matters relating to research at the site at which you are participating, the details of the local site complaints person are:

**Complaints contact person**

| Name | *[Name]* |
| --- | --- |
| Position | *[Position]* |
| Telephone | *[Phone number]* |
| Email | *[Email address]* |

If you have any complaints about any aspect of the study, the way it is being conducted or any questions about being a research participant in general, you can contact:

| Reviewing HREC name | Peter MacCallum Cancer Centre HREC |
| --- | --- |
| HREC Executive Officer | Ethics Coordinator |
| Telephone | (03) 8559 7540 |
| Email | [ethics@petermac.org](mailto:Dianne.Snowden@petermac.org) |

**Reviewing HREC approving this research** **and HREC Executive Officer details**

**Local HREC Office contact (Single Site - Research Governance Officer)**

| Name | *[Name]* |
| --- | --- |
| Position | *[Position]* |
| Telephone | *[Phone number]* |
| Email | *[Email address]* |

**Consent Form**

| **Title** | *Multi-Omics Evaluation of Peritoneal Fluid in Gastroesophageal Cancer (OMEGCA): A prospective trial to develop a sensitive assay to detect clinically occult peritoneal metastases* |
| --- | --- |
| **Coordinating Principal Investigator/**  **Principal Investigator** | *Dr. David Liu*  *A/Prof. Nicholas Clemons* |
| **Associate Investigator(s)**  *(if required by institution)* | *Prof. Niall Tebbutt, Prof. Jeanne Tie, Dr. Stephen Wong, Prof. David Watson, Dr. Markus Trochsler, Dr. Margaret Lee, Dr. Darren Wong, A/Prof, Ronald Ma, Dr. Zexi Allan, Dr. Krinal Mori, Dr. Nicole Winter, Dr. Sarah Martin, Dr. Geraldine Ooi, Dr. Yahya Al-Habbal, and Ms. Katheryn Hall* |
| **Location** *(where CPI/PI will recruit)* | *Peter MacCallum Cancer Centre, VIC*  *Austin Health, VIC*  *Northern Health, VIC*  *St Vincent’s Health, VIC*  *Melbourne Health, VIC*  *Western Health, VIC*  *Eastern Health, VIC*  *Monash Health, VIC*  *Flinders Medical Centre, SA*  *Royal Adelaide Hospital, SA*  *Queen Elizabeth Hospital, SA* |

**Consent Agreement**

- I have read the Participant Information Sheet or someone has read it to me in a language that I understand.
- I understand the purposes, procedures and risks of the research described in the study.
- I have had an opportunity to ask questions and I am satisfied with the answers I have received.
- I freely agree to participate in this research study as described and understand that I am free to withdraw at any time during the study without affecting my future health care.
- I understand that I will be given a signed copy of this document to keep.
- I give permission for my doctors, other health professionals, hospitals or laboratories outside this hospital to release information to *Peter MacCallum Cancer Centre* concerning my condition and treatment for the purposes of this study. I understand that such information will remain confidential.

**Please indicate:**

I consent to the use of my coded information and leftover samples for future research which includes genetic testing.

YES □ NO □

I wish to be contacted if findings are made that have implications for me or my family YES □ NO □

*If yes, please indicate*:

If I am unable to be contacted, I consent to this information being given to a member of my family

YES □ NO □

*If yes, please complete:*

Name of family member:

Relationship to participant:

Address:

Phone number:

**Declaration by Participant – for participants who have read the information**

|  | | | | | | |
| --- | --- | --- | --- | --- | --- | --- |
|  | Name of Participant (please print) | |  |  |  |  |
|  | | | | | | |
|  | Signature |  | | Date |  |  |
|  | | | | | | |

**Declaration - for participants unable to read the information and consent form**

| Witness to the informed consent process  Name (please print) __________________________________________________________  Signature _______________________________ Date ______________________________  * Witness is not to be the investigator, a member of the study team or their delegate. In the event that an interpreter is used, the interpreter may not act as a witness to the consent process. Witness must be 18 years or older. |
| --- |

**Declaration by Study Doctor/Senior Researcher^†^**

I have given a verbal explanation of the research study, its procedures and risks and I believe that the participant has understood that explanation.

|  | | | | | | |
| --- | --- | --- | --- | --- | --- | --- |
|  | Name of Study Doctor/  Senior Researcher^†^ (please print) | |  | | |  |
|  | | | | | |  |
|  | Signature |  | | Date |  |  |
|  | | | | | | |

^†^ A senior member of the research team must provide the explanation of, and information concerning, the research study.

Note: All parties signing the consent section must date their own signature.

**Form for Withdrawal of Participation**

| **Title** | *Multi-Omics Evaluation of Peritoneal Fluid in Gastroesophageal Cancer (OMEGCA): A prospective trial to develop a sensitive assay to detect clinically occult peritoneal metastases* |
| --- | --- |
| **Coordinating Principal Investigator/**  **Principal Investigator** | *Dr. David Liu*  *A/Prof. Nicholas Clemons* |
| **Associate Investigator(s)**  *(if required by institution)* | *Prof. Niall Tebbutt, Prof. Jeanne Tie, Dr. Stephen Wong, Prof. David Watson, Dr. Markus Trochsler, Dr. Margaret Lee, Dr. Darren Wong, A/Prof, Ronald Ma, Dr. Zexi Allan, Dr. Krinal Mori, Dr. Nicole Winter, Dr. Sarah Martin, Dr. Geraldine Ooi, Dr. Yahya Al-Habbal, and Ms. Katheryn Hall* |
| **Location** *(where CPI/PI will recruit)* | *Peter MacCallum Cancer Centre, VIC*  *Austin Health, VIC*  *Northern Health, VIC*  *St Vincent’s Health, VIC*  *Melbourne Health, VIC*  *Western Health, VIC*  *Eastern Health, VIC*  *Monash Health, VIC*  *Flinders Medical Centre, SA*  *Royal Adelaide Hospital, SA*  *Queen Elizabeth Hospital, SA* |

**Please indicate:**

I wish to withdraw from participation in the optional future research and understand that such withdrawal will not affect my routine treatment, my relationship with those treating me or my relationship with [institution]. YES □ NO □

I wish for my samples to be kept and analysed or destroyed?

Kept and analysed □ Destroyed □

**Declaration by Participant**

I wish to withdraw from participation in the above research study including withdrawal of consent to use samples and data collected for research, and understand that such withdrawal will not affect my routine treatment, my relationship with those treating me or my relationship with *[Institution]*.

|  | | | | | | |
| --- | --- | --- | --- | --- | --- | --- |
|  | Name of Participant (please print) | |  |  |  |  |
|  | | | | | | |
|  | Signature |  | | Date |  |  |
|  | | | | | | |

*In the event that the participant’s decision to withdraw is communicated verbally, the Study Doctor/Senior Researcher will need to provide a description of the circumstances below.*

|  |
| --- |

**Declaration by Study Doctor/Senior Researcher^†^**

I have given a verbal explanation of the implications of withdrawal from the research study and I believe that the participant has understood that explanation.

|  | | | | | | |
| --- | --- | --- | --- | --- | --- | --- |
|  | Name of Study Doctor/  Senior Researcher^†^ (please print) | |  | | |  |
|  | | | | | |  |
|  | Signature |  | | Date |  |  |
|  | | | | | | |

^†^ A senior member of the research team must provide the explanation of and information concerning withdrawal from the research study.

Note: All parties signing the consent section must date their own signature.
