# Supplementary material for: Multi-omics evaluation of peritoneal fluid in gastroesophageal cancer (OMEGCA): protocol for a prospective multicentre cohort study to detect occult peritoneal metastases in patients undergoing curative-intent treatment
Source: PLoS One. 2025 Apr 16;20(4):e0318615. doi: 10.1371/journal.pone.0318615 (PMC12002517; doi:10.1371/journal.pone.0318615)

| protocol |
| --- |
| 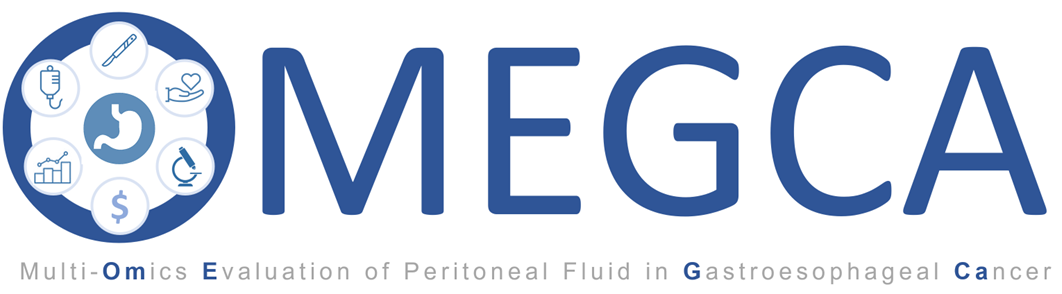  A prospective trial to develop a sensitive assay to detect clinically occult peritoneal metastases |
| **PROTOCOL NUMBER**  Version: 2.0  Date: 03/04/2024  **COORDINATING PRINCIPAL INVESTIGATORS**  Dr. David Liu  A/Prof. Nicholas Clemons  **STUDY SPONSOR**  Peter MacCallum Cancer Centre |
|  |
|  |
| **CONFIDENTIAL**  This document is confidential and the property of the Peter MacCallum Cancer Centre. No part of it may be transmitted, reproduced, published, or used without prior written authorization from the sponsor.  **STATEMENT OF COMPLIANCE**  This document is a protocol for a research project. This study will be conducted in compliance with all stipulation of this protocol, the conditions of the ethics committee approval, the NHMRC National Statement on ethical Conduct in Human Research (2007) and the Note for Guidance on Good Clinical Practice (CPMP/ICH-135/95). |

# Table of Contents

[Signature Page 4](#_Toc157143415)

[STUDY SYNOPSIS 5](#_Toc157143416)

[**1.** **Glossary of Abbreviations & Terms** 8](#_Toc157143417)

[**2.** **Study Sites and Investigators** 9](#_Toc157143418)

[a. Study Investigators 9](#_Toc157143419)

[b. Study Location/s 10](#_Toc157143420)

[**3.** **Introduction/Background Information** 11](#_Toc157143421)

[a. Lay Summary 11](#_Toc157143422)

[b. Introduction 11](#_Toc157143423)

[c. Background information 12](#_Toc157143444)

[**4.** **Study Objectives** 15](#_Toc157143445)

[a. Hypothesis 15](#_Toc157143446)

[b. Study Objectives 15](#_Toc157143447)

[c. Study Endpoints 15](#_Toc157143449)

[**5.** **Study Design** 17](#_Toc157143450)

[a. Study Type, Design, Schedule & Methodology 17](#_Toc157143451)

[b. Standard Care and Additional to Standard Care Procedures 22](#_Toc157143452)

[**6.** **Study Population** 24](#_Toc157143453)

[a. Recruitment Procedure 24](#_Toc157143454)

[b. Inclusion Criteria 24](#_Toc157143455)

[c. Exclusion Criteria 24](#_Toc157143456)

[d. Consent 24](#_Toc157143457)

[**7.** **Participant Safety and Withdrawal** 26](#_Toc157143458)

[a. Risk Management and Safety 26](#_Toc157143459)

[b. Handling of Withdrawals 27](#_Toc157143460)

[c. Replacements 27](#_Toc157143461)

[**8.** **Statistical Methods** 28](#_Toc157143462)

[a. Sample Size Estimation & Justification & Power Calculation 28](#_Toc157143463)

[b. Statistical Methods To Be Undertaken 28](#_Toc157143464)

[**9.** **Storage of Blood and Tissue Samples** 29](#_Toc157143465)

[a. Details of where samples will be stored, and the type of consent for future use of samples 29](#_Toc157143466)

[**10.** **Data Security & Handling** 30](#_Toc157143467)

[a. Details of where records will be kept & How long will they be stored 30](#_Toc157143468)

[b. Confidentiality and Security 30](#_Toc157143469)

[**11.** **Results, Outcomes & Future Plans** 31](#_Toc157143470)

[a. Results and reporting of outcomes 31](#_Toc157143471)

[b. Additional studies 31](#_Toc157143472)

[c. Project closure processes 31](#_Toc157143473)

[**12.** **Investigator’s responsibilities** 32](#_Toc157143538)

[**13.** **Criteria for centre inclusion within OMEGCA** 33](#_Toc157143539)

[**14.** **Authorship for publications** 33](#_Toc157143540)

[**15.** **Exclusion from study** 33](#_Toc157143542)

[**16.** **Modes of communication** 34](#_Toc157143543)

[**17.** **References** 35](#_Toc157143544)

[**18.** **Appendix 1 – ECOG performance scale** 37](#_Toc157143690)

# Signature Page

**Sponsor Approval**

The undersigned confirms that the following protocol has been accepted and approved by the sponsor:

|  | | |
| --- | --- | --- |
| Signature |  | Date |
|  | | |
| Name (please print) |  |  |
|  | | |
| Position |  |  |

**Principal Investigator Agreement**

I have read and understood all sections of the protocol (including other manuals and documents referenced to in the protocol).

I agree to conduct the trial in compliance with the approved protocol, the principles outlined in the International Council on Harmonisation/Good Clinical Practice (ICH/CGP) and in compliance with all applicable local laws and regulatory requirements.

I agree to report all information and data in accordance with the protocol, including reporting any serious adverse events as outlined in the protocol.

I agree to ensure that the confidential information contained in this document will not be used for any other purpose other than the evaluation or conduct of the trial without the prior written consent of the sponsor.

|  | | |
| --- | --- | --- |
| Signature |  | Date |
|  | | |
| Name (please print) |  | Site |

| STUDY SYNOPSIS |  |
| --- | --- |

| **Title:** | Multi-**Om**ics **E**valuation of Peritoneal Fluid in **G**astroesophageal **Ca**ncer (**OMEGCA**): A prospective trial to develop a sensitive assay to detect clinically occult peritoneal metastases |
| --- | --- |
| **Short Title:** | Multi-**Om**ics **E**valuation of Peritoneal Fluid in **G**astroesophageal **Ca**ncer (**OMEGCA**) |
| **Design:** | OMEGCA will be a prospective non-interventional and translational cohort study enrolling patients undergoing routine peritoneal lavage cytology (PLC) and subsequent curative-intent treatment (either upfront surgery or perioperative systemic treatment and surgery) for gastroesophageal cancer at hospitals across Victoria and South Australia.  Tumour biopsies, blood and peritoneal lavage fluid will be collected during routine pre-treatment PLC for all patients. A subset of patients will have a blood and peritoneal lavage fluid collected at the time of surgical resection, and blood collected at the first post-surgical follow-up clinic.  These samples will be centralised to a research laboratory at the Peter MacCallum Cancer Centre for processing and analysis. Part of the collected biospecimens will undergo methylomic analysis at the Peter MacCallum Cancer Centre, VIC. The remaining biospecimens will undergo genomic whole exome sequencing analysis at the Johns Hopkins University Hospital, and Haystack Oncology, USA. Methylomic and genomic analyses are aimed at detecting the presence of tumour derived DNA in peritoneal fluid (ptDNA). ptDNA status will be correlated to different clinical outcomes including patient survival.  Recruitment and sample analysis will be conducted over 2 years with clinical follow-up extended to 5 years from the last patient to capture disease free and overall survival data.  The final analytical cohort will include at least 200 patient samples. Part of this cohort will include around 100 patient samples and clinical data already collected as part of a pre-existing Peter Mac led translational project entitled ‘*Improving outcomes in gastroesophageal cancer*’ (HREC/44873/PMCC-2018, Project No: 18/211), which has a compatible study design to this project. |
| **Lead Study Centres:** | Peter MacCallum Cancer Centre |
| **Participating Hospitals:** | 10 to 12 sites |
| **Primary Objectives:** | 1. To determine whether pre-treatment ptDNA, detected using genomic and methylomic approaches, predicts disease free survival (DFS) in gastroesophageal cancer patients |
| **Secondary Objectives** | 1. To analyse the ability of methylomic and genomic assays, in comparison and in combination, to detect ptDNA and predict DFS and overall survival (OS). 2. To compare the performance of ptDNA vs. conventional staging methods (PLC, PET/CT) to predict histopathological features of the tumour, patterns of disease recurrence, DFS and OS. |
| **Exploratory Objectives** | 1. To compare the cost-effectiveness of genomic vs. methylomic approaches to detect ptDNA to inform translation into clinical practice 2. To compare ptDNA detection rates before (at time of PLC) and after (at time of surgical resection) neoadjuvant therapy. 3. To curate and catalogue actionable molecular targets identified from whole exome sequencing of peritoneal fluid to inform future clinical trials. 4. To compare ptDNA versus circulating tumour DNA (i.e. plasma) to predict sites and patterns of disease recurrence. |
| **Inclusion Criteria:** | 1. All patients ≥18 years-of-age at time of signing consent 2. Have a histologically confirmed diagnosis of one of the following:    1. Gastric adenocarcinoma    2. Gastroesophageal junction adenocarcinoma 3. Is undergoing staging laparoscopy and peritoneal lavage cytology 4. Provides informed consent |
| **Exclusion Criteria:** | 1. Strictly oesophageal cancer 2. Distant organ disease on CT and PET/CT 3. Non-regional nodal disease on CT and PET/CT 4. Peritoneal carcinomatosis on CT and PET/CT 5. Performance status ECOG ≥3 6. Undergoing emergency surgery 7. Non-curative/palliative-intent treatment |
| **Sample Size:** | At least 200 patients |
| **Primary endpoint** | 2-year DFS |
| **Secondary endpoints** | - *Survival*: 3-year peritoneal DFS, 3-year overall survival (OS), 5-year DFS, and 5-year OS - *Peritoneal lavage cytology status*: cytology present, absent/indeterminant - *Imaging (CT/PET scans) and endoscopy*: Location of disease recurrence - *Primary tumour histopathological features*: T-stage, N-stage, grade, lymphovascular invasion, perineural infiltration, histological subtypes, tumour size, and tumour regression grading - *Cost effectiveness analysis*: accounting for assay performance, direct-costs, technical aspects, clinical impact, and quality-adjusted life years. - *Genomic analysis of ptDNA*: mutant allelic frequency thresholds, presence of actionable therapeutic targets against a registry of clinically proven and experimental therapies. |
| **Statistical Methods:** | Power calculation was performed to compare subgroups 1 and 2 (i.e., ptDNA-positive/PLC-negative vs. ptDNA-negative/PLC-negative cohort) with respect to the primary endpoint of this study.  The clinical utility of ptDNA (ptDNA-positive vs ptDNA-negative), as determined by genomic and methylomic output, will be evaluated in consultation with our study biostatistician using the following metrics: sensitivity, specificity, positive-predictive value, negative-predictive value, receiver operator characteristics, Kaplan Meier and Ccox regression, against the primary and secondary endpoints. Co-variates in these analyses will be accounted for using hierarchical multi-variate logistic regression algorithms. Cost-effectiveness analysis will be undertaken together with a clinical costing analyst. |

## **Glossary of Abbreviations & Terms**

| **Abbreviation** | **Description (using lay language)** |
| --- | --- |
| AJCC 8^th^ edition | American Joint Committee on Cancer, 8^th^ edition staging criteria for cancer |
| CT | Computer tomography scan |
| ctDNA | Circulating tumour DNA |
| DFS | Disease free survival |
| DNA | Deoxyribonucleic acid |
| ECOG grade | European Clinical Oncology Group grading of performance status |
| GOJ | Gastroesophageal junction |
| OS | Overall survival |
| PLC | Peritoneal lavage cytology |
| ptDNA | Peritoneal tumour DNA |
| PET | Positron Emission Tomography scan |

## **Study Sites and Investigators**

### Study Investigators

**COORDINATING PRINCIPAL INVESTIGATORS**

| **Name**: | Dr. David Liu | **Name**: | A/Prof. Nicholas Clemons |
| --- | --- | --- | --- |
| **Role**: | Surgeon | **Role**: | Chief scientist, Lab head |
| **Institute**: | Peter MacCallum Cancer Centre | **Institute**: | Peter MacCallum Cancer Centre |
| **Address**: | 305 Grattan Street, Melbourne, 3000, VIC | **Address**: | 305 Grattan Street, Melbourne, 3000, VIC |
| **Phone**: | 0402 857 529 | **Phone**: | 0408 554 234 |
| **Email**: | [David.Liu@petermac.org](mailto:David.Liu@petermac.org) | **Email**: | [Nicholas.Clemons@petermac.org](mailto:Nicholas.Clemons@petermac.org) |

**CO-INVESTIGATORS**

| **Name**: | Prof. Niall Tebbutt | **Name**: | Prof. Jeanne Tie |
| --- | --- | --- | --- |
| **Role**: | Gastrointestinal oncologist | **Role**: | Gastrointestinal oncologist, Clinical and transitional expert, liquid biopsy expert |
| **Institute**: | Olivia Newton-John Cancer Centre | **Institute**: | Peter MacCallum Cancer Centre |
| **Address**: | 145 Studley Road, Heidelberg, 3084, VIC | **Address**: | 305 Grattan Street, Melbourne, 3000, VIC |
| **Phone**: | 0410 582 006 | **Phone**: | 0420 757 532 |
| **Email**: | [niall.tebbutt@austin.org.au](mailto:niall.tebbutt@austin.org.au) | **Email**: | [Jeanne.Tie@petermac.org](mailto:Jeanne.Tie@petermac.org) |

| **Name**: | Dr. Stephen Wong | **Name**: | Prof. David Watson |
| --- | --- | --- | --- |
| **Role**: | Scientist, Lab head | **Role**: | Director, Clinical Trials Network ANZ |
| **Institute**: | Peter MacCallum Cancer Centre | **Institute**: | Flinders Medica Centre |
| **Address**: | 305 Grattan Street, Melbourne, 3000, VIC | **Address**: | Flinders Drive, Bedford Park, 5042, SA |
| **Phone**: | 03 8559 9342 | **Phone**: | 0410 506 244 |
| **Email**: | [Stephen.Wong@petermac.org](mailto:Stephen.Wong@petermac.org) | **Email**: | [david.watson@flinders.edu.au](mailto:david.watson@flinders.edu.au) |

| **Name**: | Dr. Markus Trochsler | **Name**: | Dr. Margaret Lee |
| --- | --- | --- | --- |
| **Role**: | Surgeon | **Role**: | Gastrointestinal oncologist |
| **Institute**: | Royal Adelaide Hospital | **Institute**: | Eastern Health |
| **Address**: | Port Road, Adelaide, 5000, SA | **Address**: | 8 Arnold Street, Box Hill, 3128, VIC |
| **Phone**: | 0427 276 157 | **Phone**: | 0403 007 320 |
| **Email**: | [markus.trochsler@sa.gov.au](mailto:markus.trochsler@sa.gov.au) | **Email**: | [margaret.lee@monash.edu](mailto:margaret.lee@monash.edu) |

| **Name**: | Dr. Darren Wong | **Name**: | A/Prof. Ronald Ma |
| --- | --- | --- | --- |
| **Role**: | Biostatistician | **Role**: | Cost analyst |
| **Institute**: | Austin Health | **Institute**: | Austin Health |
| **Address**: | 145 Studley Road, Heidelberg, 3084, VIC | **Address**: | 145 Studley Road, Heidelberg, 3084, VIC |
| **Phone**: | 0438 793 388 | **Phone**: | 03 9496 5000 |
| **Email**: | [Darren.WONG@austin.org.au](mailto:Darren.WONG@austin.org.au) | **Email**: | [ronald.ma@austin.org.au](mailto:ronald.ma@austin.org.au) |

| **Name**: | Dr. Zexi Allan | **Name**: | Dr. Krinal Mori |
| --- | --- | --- | --- |
| **Role**: | Laboratory researcher | **Role**: | Surgeon |
| **Institute**: | Peter MacCallum Cancer Centre | **Institute**: | Northern Health |
| **Address**: | 305 Grattan Street, Melbourne, 3000, VIC | **Address**: | 185 Cooper Street, Epping, 3076, VIC |
| **Phone**: | 0420 853 245 | **Phone**: | 0433 697 718 |
| **Email**: | [zexi.allan@petermac.org](mailto:zexi.allan@petermac.org) | **Email**: | [Krinal.Mori@nh.org.au](mailto:Krinal.Mori@nh.org.au) |

| **Name**: | Dr. Nicole Winter | **Name**: | Dr Sarah Martin |
| --- | --- | --- | --- |
| **Role**: | Surgeon | **Role**: | Surgeon |
| **Institute**: | Melbourne Health | **Institute**: | Monash Health |
| **Address**: | 300 Grattan Street, Parkville, 3052, VIC | **Address**: | 246 Clayton Road, Clayton, 3168, VIC |
| **Phone**: | 0414 642 653 | **Phone**: | 03 9594 6666 |
| **Email**: | [Nicole.Winter2@mh.org.au](mailto:Nicole.Winter2@mh.org.au) | **Email**: | [Sarah.Martin@monashhealth.org](mailto:Sarah.Martin@monashhealth.org) |

| **Name**: | Ms. Katheryn Hall | **Name**: | Dr. Geraldine Ooi |
| --- | --- | --- | --- |
| **Role**: | Study coordinator | **Role**: | Surgeon |
| **Institute**: | Peter MacCallum Cancer Centre | **Institute**: | Monash Health |
| **Address**: | 305 Grattan Street, Melbourne, 3000, VIC | **Address**: | 246 Clayton Road, Clayton, 3168, VIC |
| **Phone**: | 0419 557 264 | **Phone**: | 0411 113 673 |
| **Email**: | [Katheryn.Hall@petermac.org](mailto:Katheryn.Hall@petermac.org) | **Email**: | [geraldine.ooi@monash.edu](mailto:geraldine.ooi@monash.edu) |

| **Name**: | Dr. Yahya Al-Habbal |
| --- | --- |
| **Role**: | Surgeon |
| **Institute**: | Western Health |
| **Address**: | 160 Gordon Street, Footscray, 3011, VIC |
| **Phone**: | 0409 942 002 |
| **Email**: | [Yahya.Al-Habbal@wh.org.au](mailto:Yahya.Al-Habbal@wh.org.au) |

### Study Location/s

| **Site** | **Address** | **Contact Person** | **Phone** | **Email** |
| --- | --- | --- | --- | --- |
| Peter MacCallum Cancer Centre | 305 Grattan Street, Melbourne, 3000, VIC | A/Prof Nicholas Clemons | 0408 554 234 | [Nicholas.Clemons@petermac.org](mailto:Nicholas.Clemons@petermac.org) |
| Austin Health | 145 Studley Road, Heidelberg, 3084, VIC | Dr David Liu | 0402 857 529 | [David.Liu2@austin.org.au](mailto:David.Liu2@austin.org.au) |
| Northern Health | 185 Cooper Street, Epping, 3076, VIC | Dr Krinal Mori | 0433 697 718 | [Krinal.Mori@nh.org.au](mailto:Krinal.Mori@nh.org.au) |
| St Vincent’s Hospital | 41 Victoria Parade, Fitzroy, 3065, VIC | Dr Nicole Winter | 0414 642 653 | [Nicole.Winter2@mh.org.au](mailto:Nicole.Winter2@mh.org.au) |
| Melbourne Health | 300 Grattan Street, Parkville, 3052, VIC | Dr Nicole Winter | 0414 642 653 | [Nicole.Winter2@mh.org.au](mailto:Nicole.Winter2@mh.org.au) |
| Western Health | 160 Gordon Street, Footscray, 3011, VIC | Dr Yahya Al-Habbal | 0409 942 002 | [Yahya.Al-Habbal@wh.org.au](mailto:Yahya.Al-Habbal@wh.org.au) |
| Eastern Health | 8 Arnold Street, Box Hill, 3128, VIC | Dr Margaret Lee | 0403 007 320 | [margaret.lee@monash.edu](mailto:margaret.lee@monash.edu) |
| Monash Health | 246 Clayton Road, Clayton, 3168, VIC | Dr Sarah Martin | 03 9594 6666 | [Sarah.Martin@monashhealth.org](mailto:Sarah.Martin@monashhealth.org) |
| Flinders Medical Centre | Flinders Drive, Bedford Park, 5042, SA | Prof David Watson | 0410 506 244 | [david.watson@flinders.edu.au](mailto:david.watson@flinders.edu.au) |
| Royal Adelaide Hospital | Port Road, Adelaide, 5000, SA | Dr Markus Trochsler | 0427 276 157 | [markus.trochsler@sa.gov.au](mailto:markus.trochsler@sa.gov.au) |
| Queen Elizabeth Hospital | 28 Woodville Road, Woodville, 5011, SA | Dr Markus Trochsler | 0427 276 157 | [markus.trochsler@sa.gov.au](mailto:markus.trochsler@sa.gov.au) |

## **Introduction/Background Information**

### Lay Summary

The staging of stomach and oesophageal cancer is crucial for determining whether this disease is curable or not. This information allows patients and doctors to select the right treatment approach. Stomach and oesophageal cancers have a high likelihood of spreading to the abdominal cavity (peritoneum). The presence of peritoneal disease is currently considered incurable. Unfortunately, despite our best staging tests (CT and PET scans, and peritoneal washings to detect cancer cells), our ability to accurately stage the peritoneum is inadequate. This means that many patients are being offered aggressive and morbid treatments with potentially little benefit. Therefore, an accurate test to stage the peritoneum is urgently needed to personalise treatment and avoid over-treating patients with stomach and oesophageal cancer. ptDNA is cancer-derived DNA detectable in peritoneal washings. As this is a molecular test, we propose that ptDNA detection should be more accurate than current methods to stage the peritoneum. In our laboratory, we have developed the assays to detect ptDNA.

In the OMEGCA study (Multi-Omics Evaluation of Peritoneal Fluid in Gastroesophageal Cancer), we will recruit at least 200 patients with stomach and oesophageal cancer, and test their peritoneal washings, collected as part of routine staging, for ptDNA. We will compare the performance of two different methods to detect ptDNA, and determine whether ptDNA positivity correlates with patient survival, tumour characteristics and patterns of cancer recurrence.

If successful, this study will produce a novel and accurate molecular test to detect microscopic peritoneal cancer deposits. This information will improve disease prognostication, facilitate patient counselling, inform clinical decision-making, and personalise cancer treatment to maximise benefit and reduce harm.

### Introduction

In Australia, gastroesophageal cancer patients have a dismal 5-year survival rate between 20%-35%.^1, 2^ Accurate cancer staging is the cornerstone for determining therapeutic intent and optimal multidisciplinary management. Gastroesophageal cancers have a high propensity to spread into the peritoneal cavity, and such patients are currently considered incurable. Therefore, *early detection of peritoneal micro-metastases alters therapeutic intent, providing a vital opportunity to personalise treatment for patients.*

Currently, our ability to accurately stage the peritoneum is poor. The current “best-practice” staging methods of CT/PET imaging and peritoneal lavage cytology (PLC) *miss peritoneal disease in a substantial proportion of patients,* who are subsequently offered morbid and ineffective treatments. A sensitive and specific assay to accurately stage the peritoneum is urgently needed to guide therapeutic management and improve survival outcomes of patients with gastroesophageal cancer.

Peritoneal tumour DNA (ptDNA) is tumour-derived DNA detectable in peritoneal fluid. The presence of ptDNA indicates the existence of cancer cells within the peritoneum despite a negative or indeterminant CT/PET or PLC result. Thus, ptDNA detection should be more accurate than current methods of peritoneal staging. As proof-of-concept, using next generation sequencing, we found the presence of ptDNA in 4 of 11 patients (HREC/44873/PMCC-2018, Project No: 18/211), including those with undetectable metastases on CT/PET and PLC investigations.

### Background information

ACCURATE STAGING IS CRITICAL FOR TREATING GASTROESOPHAGEAL CANCER

The incidence of gastroesophageal cancer has risen markedly and now ranks as the 4^th^ most common cause of cancer-related deaths in the world.^3^ It is a lethal diagnosis with a 5-year survival of 20-35%, and of greatest concern is the rising incidence in young adults (aged <50 years).^1, 2^ Accurate staging of gastroesophageal cancer is crucial for determining prognosis, treatment intent and approach. Staging typically involves endoscopy, PET/CT imaging and peritoneal lavage cytology (PLC). PLC involves irrigating the peritoneal cavity with saline and retrieving this fluid to assess for the presence of cancer cells. Patients without demonstrable distant organ or peritoneal disease (~40%) are treated with chemotherapy and radical surgery in the belief that aggressive and *potentially morbid* treatment will deliver a cure. However, the vast majority of these patients have locally advanced disease which carries a high risk for trans-coelomic spread into the peritoneum. Unfortunately, the presence of peritoneal metastases is currently considered incurable.^4^ Therefore, early and accurate detection of peritoneal disease, especially micro-metastases, is critical as it alters treatment intent and approach, either through avoiding morbid and futile treatments or enrolling into clinical trials such as studies evaluating peritoneal-directed therapy.

CLINICAL URGENCY: OUR ABILITY TO ACCURATELY STAGE THE PERITONEUM IS POOR

Currently, conventional staging methods are unreliable in identifying peritoneal disease. For example, CT/PET imaging is unable to detect peritoneal disease unless advanced features (e.g. nodules or ascites) are present.^5^ Despite the pervasive use of PLC, its sensitivity for detecting micro-metastases ranges from 10-80%, with 20-40% of tests returning an indeterminant result.^6^ Moreover, newer technologies such as testing circulating tumour DNA in blood does not accurately stage the peritoneum.^7^ As a result, 50% of patients who undergo aggressive treatment with curative-intent, develop early recurrence within 11 months after radical surgery,^8, 9^ and over 50% of these patients recur in the peritoneum as their first site.^10^ This suggests that *peritoneal micro-metastases were present and missed at the time of staging* and patients have consequently undergone highly morbid treatment for little benefit. Therefore, a sensitive assay to accurately stage the peritoneum is urgently needed to enable the appropriate personalise therapeutic management of patients with gastroesophageal cancer, and thus improve survival and quality-of-life outcomes.

POTENTIAL SOLUTION: PERITONEAL TUMOUR DNA AS A NOVEL BIOMARKER OF PERITONEAL DISEASE

| 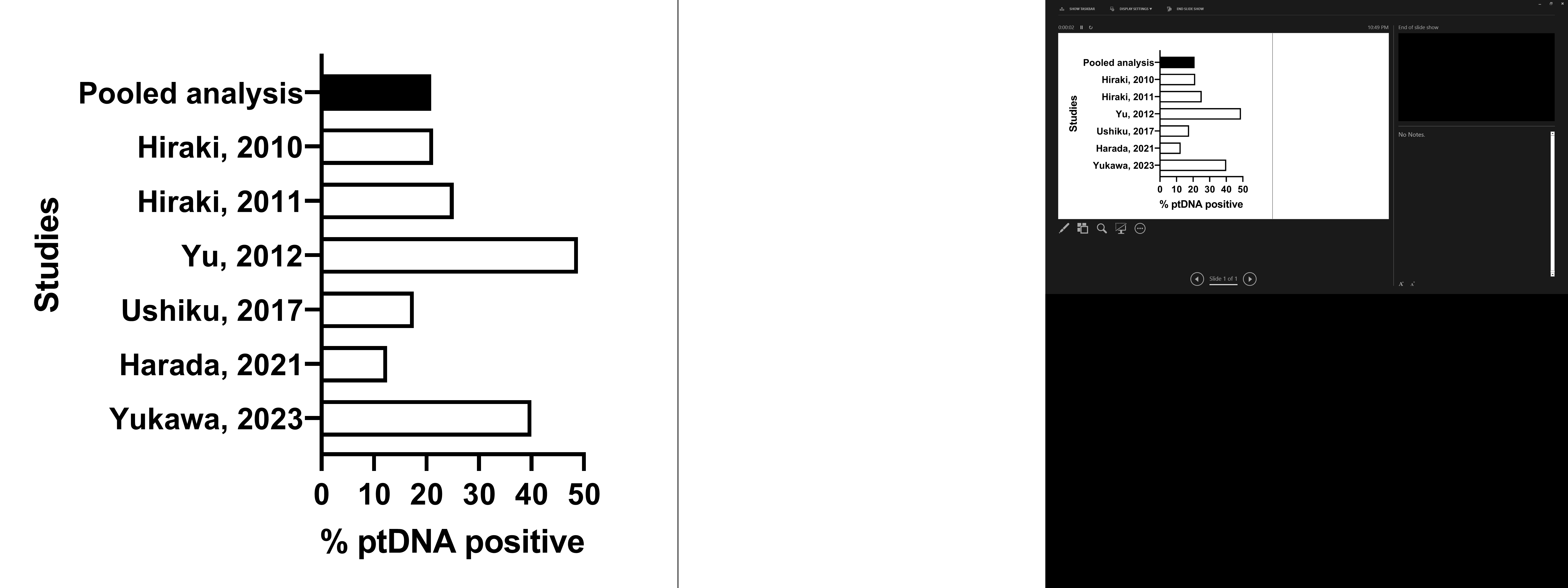  **Figure 1.** *ptDNA positivity in 6 studies* | Peritoneal tumour DNA (ptDNA) is tumour-derived DNA detectable in either the cellular or cell-free fractions of peritoneal fluid. The presence of ptDNA indicates the existence of cancer cells within the peritoneum despite a negative or indeterminant CT/PET or PLC result.^11^ Accordingly, ptDNA identified in peritoneal lavage fluid should be more sensitive than cytology in staging the peritoneum. As proof-of-concept, we performed a systematic review to examine the potential clinical utility of ptDNA in gastroesophageal cancer. From six available studies,^11-16^ we found that 1) ptDNA is detectable in peritoneal lavage fluid (*Figure 1*), 2) ptDNA may be more sensitive than PLC in detecting peritoneal metastases, and 3) ptDNA positivity can predict patient survival (*Figure 2*). |
| --- | --- |


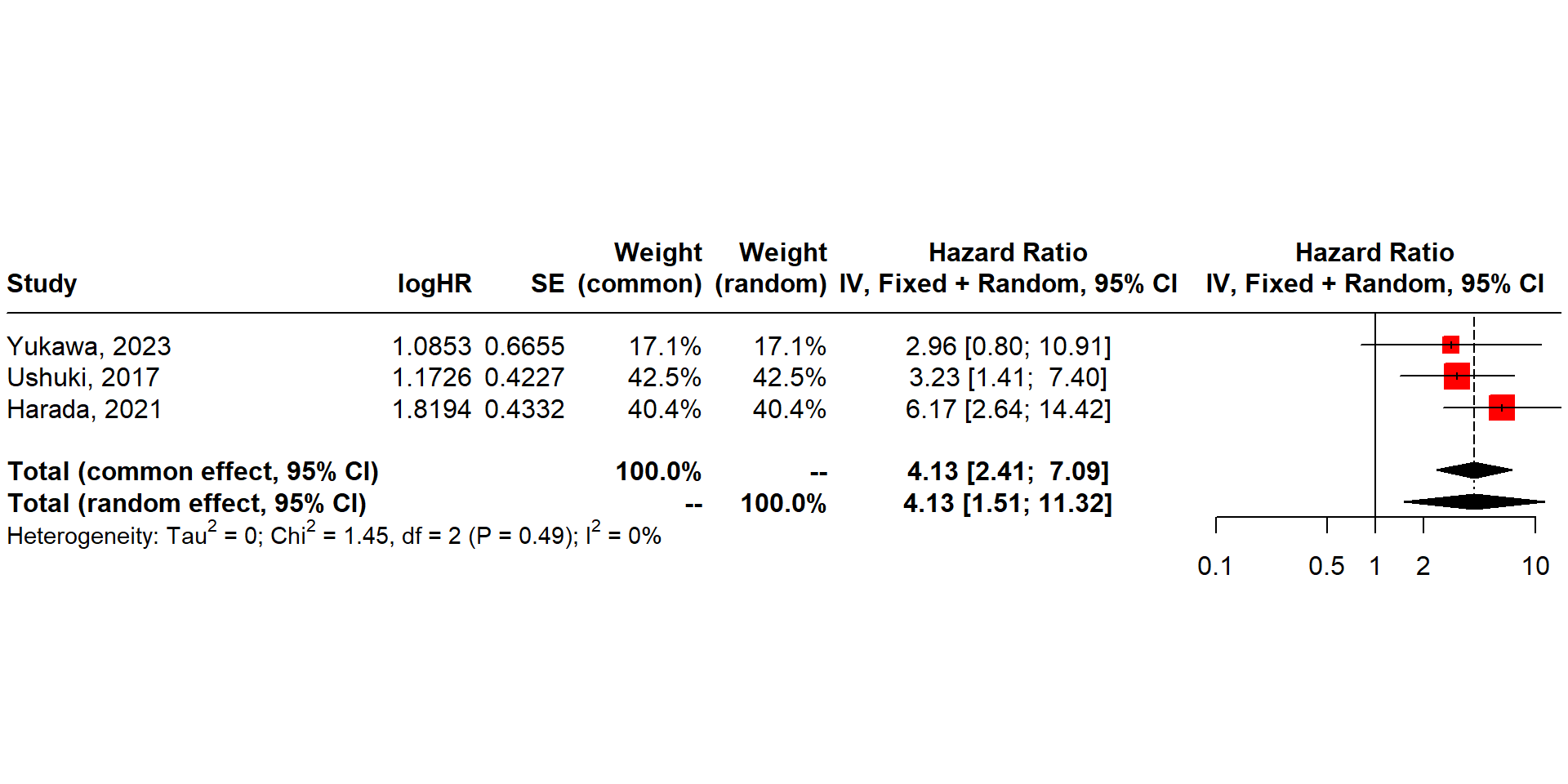


**Figure 2.** *Meta-analysis of 3 studies demonstrating that ptDNA positivity predicts increased mortality at 3 years*

KEY LIMITATIONS OF AVAILABLE EVIDENCE

Whilst these findings are promising, they also have significant limitations. **1)** They were all single institution studies from Asia, where disease biology and treatment approaches are vastly different to Western countries.^17^ **2)** They all adopted a tumour agnostic approach, which can lead to a higher false-positive ptDNA detection rate. This is problematic as treatment-intent is contingent on the specificity of this assay. **3)** These studies captured patients across all stages of gastric cancer including those with gross peritoneal metastases, thus inflating the true sensitivity of their assay. **4)** Most studies examined only one gene, which potentially limits the sensitivity of their assay. **5)** Five of the six studies evaluated methylomic analysis of peritoneal fluid with no studies comparing the accuracy of methylomic versus genomic techniques to identify ptDNA. **6)** No study has evaluated the accuracy of combining genomic and methylomic approaches to detect ptDNA and predict clinical outcomes. Our experience suggests that a tumour-informed genomic approach provides greater specificity but may lack sensitivity, whereas a tumour-agnostic methylomic method provides greater sensitivity but may lack specificity. Therefore, both techniques may be complementary and deserves in-depth evaluation. **7)** All six studies focussed on gastric cancer, with no studies examining the role of ptDNA in gastroesophageal junction cancer despite its rapidly rising incidence throughout the world including Australia. Addressing these key shortcomings are crucial to translating ptDNA tests into clinical use in an Australian cohort.

OUR APPROACH TO DEVELOPING A NOVEL AND SENSITIVE ptDNA ASSAY

Our research team have an extensive fundamental, translational and clinical experience with applying genomic and methylomic techniques to detecting tumour DNA in blood.^18-20^ In this study, we will repurpose these technologies and harness our group’s capacity to analyse peritoneal fluid collected at staging PLC. In a pilot study, we applied our highly sensitive tumour-informed whole exome sequencing platform to analyse the primary tumour tissue and peritoneal fluid of 11 patients with gastroesophageal cancer (*Figure 3*). we found that 1) genomic mutations were detectable in all primary tumour tissues and thus can be used to inform our ptDNA assay, 2) ptDNA was detectable in 4 of 11 (36%) patients and in all 3 cases with positive cytology, thus demonstrating the sensitivity and specificity of our genomic approach. Critically, 3) ptDNA was identified in one patient who was cytology-negative (*Figure 3,* Study ID 4) suggesting that this patient had underlying peritoneal metastases that were missed using ‘current best-practice’ staging methods. This patient developed early recurrence within 9 months of ‘curative-intent’ treatment.


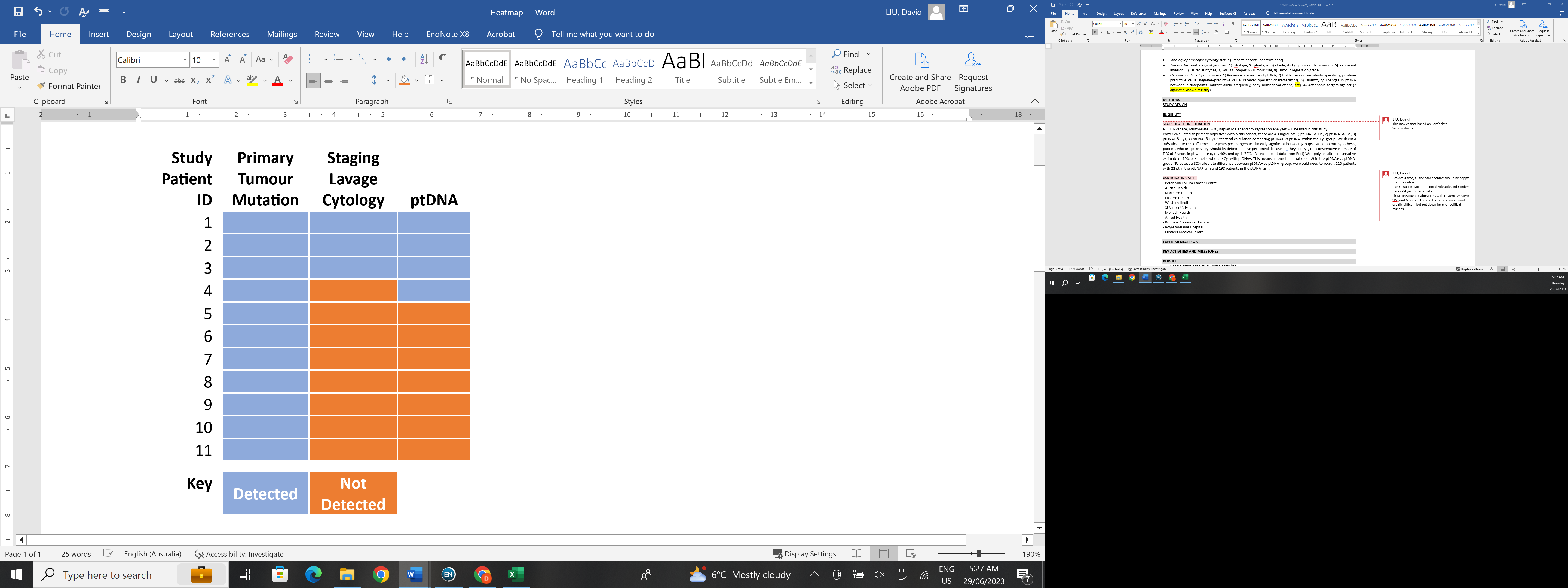


***Figure 3.*** *Pilot data using a tumour-informed whole exome sequencing platform to detect ptDNA*

PROJECT GOAL

Based on promising preliminary data, our team’s proven track record in developing clinically translatable ‘liquid biopsy’ assays,^15-17^ and our experience in leading multi-centre studies within a collaborative research network,^21^ **our goal is to develop a molecular assay to enable accurate peritoneal staging in patients with gastroesophageal cancer.** This will be achieved by using (and comparing) genomic and methylomic techniques, through tumour informed and agnostic approaches, to analyse ptDNA in peritoneal lavage fluid from patients who are undergoing curative-intent treatment in high-volume Australian centres.

## **Study Objectives**

### Hypothesis

ptDNA is a sensitive biomarker of microscopic peritoneal metastases and prognosticates disease-free-survival (DFS) and overall survival (OS) in patients with gastroesophageal cancer.

### Study Objectives

PRIMARY OBJECTIVE:

1. To determine whether pre-treatment ptDNA, detected using genomic and methylomic approaches, predicts DFS in gastroesophageal cancer patients.

SECONDARY OBJECTIVES:

1. To analyse the ability of methylomic and genomic assays, in comparison and in combination, to detect ptDNA and predict DFS and OS.
2. To compare the performance of ptDNA vs. conventional staging methods (PLC, PET/CT) to predict histopathological features of the tumour, patterns of disease recurrence, DFS and OS.

EXPLORATORY OBJECTIVES:

1. To compare the cost-effectiveness of genomic vs. methylomic approaches to detect ptDNA to inform translation into clinical practice.
2. To compare ptDNA detection rates before (at time of PLC) and after (at time of surgical resection) neoadjuvant therapy.
3. To curate and catalogue actionable molecular targets identified from whole exome sequencing of peritoneal wash fluid to inform future clinical trials.
4. To compare ptDNA versus circulating tumour DNA (i.e. plasma) to predict sites and patterns of disease recurrence.

### Study Endpoints

PRIMARY ENDPOINTS:

- 2-year disease free survival (DFS) (*Objective 1*)

SECONDARY ENDPOINTS:

- *Survival*: 3-year peritoneal DFS, 3-year overall survival (OS), 5-year DFS, and 5-year OS (*Objectives 2 & 3*)
- *Peritoneal lavage cytology status*: present/absent/indeterminant (*Objective 3*)
- *Imaging (CT/PET scans) and endoscopy*: Location of disease recurrence (*Objective 3*)
- *Primary tumour histopathological features*: T-stage, N-stage, grade, lymphovascular invasion, perineural infiltration, histological subtypes, tumour size, and tumour regression grading (*Objective 3*)
- *Cost effectiveness*: accounting for assay performance, direct-costs, technical aspects, clinical impact, and quality-adjusted life years (*Objective 4*)
- *Genomic analysis of ptDNA*: mutant allelic frequency thresholds, presence of actionable therapeutic targets against a registry of clinically proven and experimental therapies (*Objectives 5 & 6*)
- *ctDNA status*: present/absent, mutant allelic frequency threshold (*Objectives 7*)

DEFINITIONS FOR PRIMARY AND SECONDARY ENDPOINTS:

- *Disease recurrence*: recurrence of disease after surgical resection of the primary tumour, in any site, detectable on clinical, endoscopic, and/or imaging investigations.
- *Disease free survival*: Time from date of surgical resection to date of disease recurrence
- *Overall survival*: Time from date of surgical resection to date of death
- *Cancer staging*: T, N and M stage for CT/PET scans and histopathology will be according to AJCC 8^th^ Edition for Gastric and Gastroesophageal Junction Cancers

# **Study Design**

### Study Type, Design, Schedule & Methodology

STUDY TYPE:

- Prospective non-interventional cohort study
- This study will include a cohort of patients who have undergone identical biospecimen collection, clinical treatments, and follow-up from Project No: 18/211, entitled ‘*Improving outcomes in gastroesophageal cancer*’ (HREC/44873/PMCC-2018)

ABOUT PROJECT NO: 18/211:

- Relevant to this study, project No: 18/211, entitled ‘*Improving outcomes in gastroesophageal cancer*’ (HREC/44873/PMCC-2018) was in part (as it was multi-facetted with different aims), a multi-centre biospecimen (tumour and normal tissue, blood, and peritoneal fluid) and clinical data collection project.
- Project 18/211 established the collaborative research network and generated the critical pilot data (11 patients) for this proposed study. The patient cohort recruited through Project 18/211 are identical to this proposed study.
- To avoid wastage of patient samples, research cost, human resources, and time, we propose that eligible patients who have undergone identical biospecimen collection, clinical treatments, cancer surveillance and follow-up in Project 18/211 as this proposed study, to be combined for the purpose of data analysis.

STUDY PERIOD:

- Participant recruitment: 2 years
- Clinical follow-up: Follow-up for 5 years after the last enrolled patient

STUDY SAMPLE SIZE:

- Merging of participants from this study and Project No: 18/211
- Combine total participant ≥200 based on power calculation, please see below and in section 8.

STUDY POPULATION:

- All patients with gastroesophageal junction and gastric cancer
- ≥ 18 years-of-age
- Without distant organ, non-regional nodal or gross peritoneal metastases
- Undergoing staging laparoscopy with peritoneal lavage cytology (PLC)
- Undergoing curative-intent treatment (either upfront surgery or chemo/radiotherapy and surgery) for their cancer

DESIGN SUMMARY TO ACHIEVE OBJECTIVES:

- This prospective, multicentre observational cohort study will enrol patients undergoing PLC and subsequent curative-intent treatment for gastroesophageal cancer.
- Tumour and normal (stomach and oesophagus) tissue biopsies, blood, and peritoneal lavage fluid will be collected during pre-treatment staging for all patients.
- A subset of patients (those who undergo neoadjuvant chemo/radiotherapy) will have tumour tissue, blood and peritoneal lavage fluid collected at surgery.
- Where possible, blood will be collected at post-surgical review (~4-6 weeks after surgical resection) in clinics.
- These samples will be centralised to the Tumorigenesis and Cancer Therapeutics (Clemons) Research laboratory at the Peter MaCallum Cancer Centre for processing and DNA extraction.
- A portion of extracted DNA will undergo methylomics sequencing and analysis at the Peter MaCallum Cancer Centre, through the Translational Genomics Core Facility (Collaborator: Dr Stephen Wong).
- A portion of extracted tumour and peritoneal fluid DNA will undergo genomic sequencing and analysis at the Johns Hopkins University Hospital, Kimmel Cancer Centre, USA (Collaborator: Prof Bert Vogelstein).
- A portion of extracted plasma DNA will undergo genomic sequencing and analysis at Haystack Oncology ^Pty Ltd^, USA.
- Clinical data will be collected by designated surgical and medical oncology fellows already employed in clinical roles within each participating centre.
- Recruitment and sample analysis will be conducted over 2 years with subsequent routine clinical follow-up to capture 5 years of survival data.
- Methylomic and genomic data that will indicate the presence or absence of ptDNA will be correlated with DFS, OS, and other clinical, radiological and pathological endpoints.
- Please see figure 4 for further details.


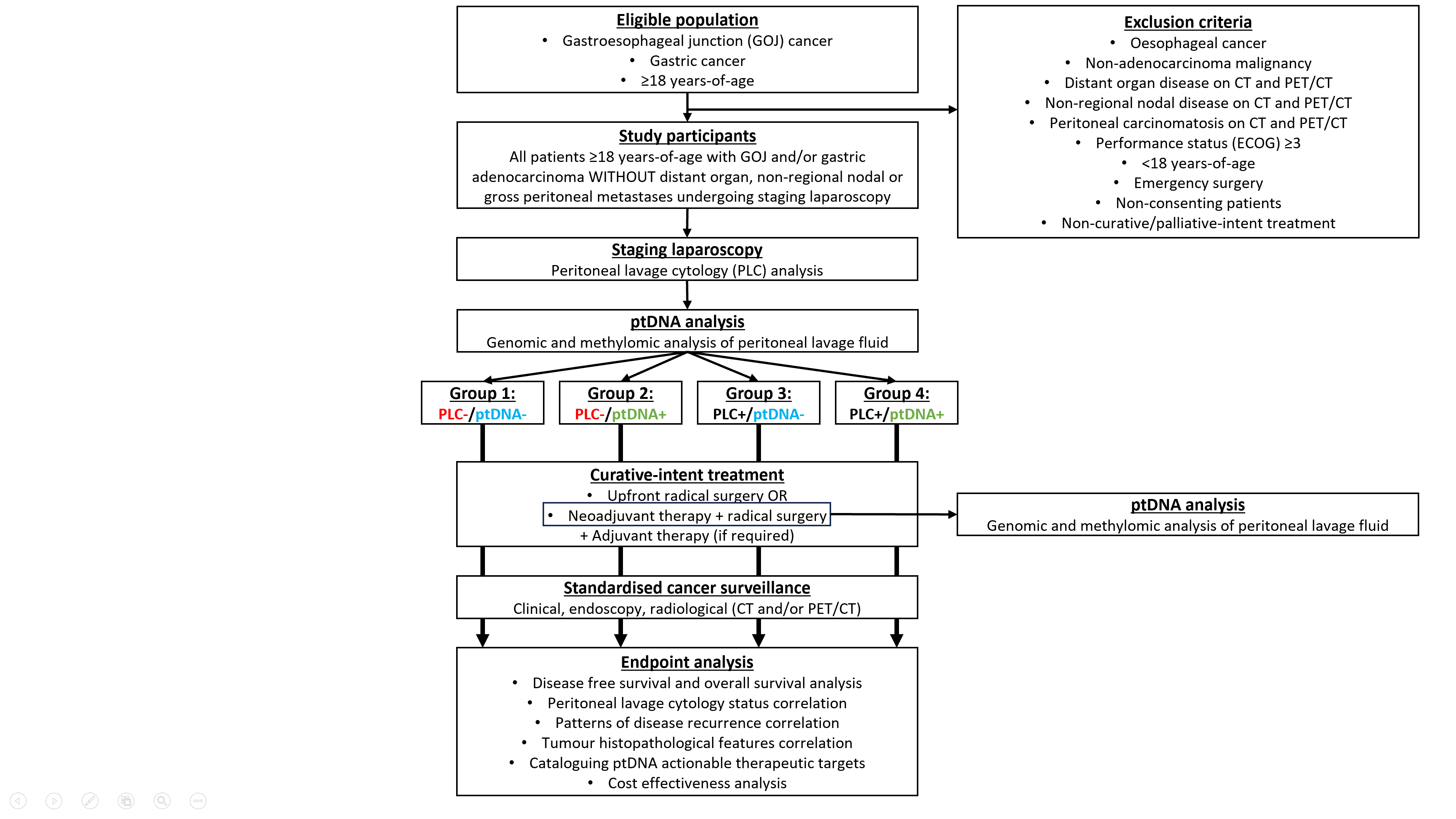


***Figure 4.*** *Schema of proposed clinical trial.*

TYPES OF COLLECTED DATA:

| **Types of data** | **Data includes** | | **Data collected as part of (source)** | **Identifiability** |
| --- | --- | --- | --- | --- |
| **Clinical data** | - Patient demography - Staging methods - Treatment details | - Surgical outcomes - Oncological outcomes | Routine care (Medical records) | Re-identifiable |
| **CT and PET data** | - Disease staging (AJCC 8^th^ edition) - Location of disease | | Routine care  (Imaging reports) | Re-identifiable |
| **Endoscopy data** | - Disease staging (AJCC 8^th^ edition) - Location of disease | | Routine care  (Endoscopy reports) | Re-identifiable |
| **Histopathological data** | - T- and N-stage (AJCC 8^th^ edition) - Tumour grade - Lymphovascular invasion | - Perineural invasion Histological subtypes - Tumour size - Regression grade - PLC cytology status | Routine care  (Pathology reports) | Re-identifiable |
| **Cost data** | - Clinical cost associated with clinical episode of care | | Routine care  (Clinical coding) | Re-identifiable |
| **Sequencing data** | - Genomic sequencing from tumour, blood, peritoneal lavage fluid - Methylomic sequencing from tumour, normal tissue, blood, peritoneal lavage fluid | | Research  (Generated by study team) | Re-identifiable |

BIOSPECIMEN COLLECTION:

Peritoneal lavage, venepuncture (for peripheral blood), endoscopy and biopsies will be performed by the clinical treating teams at each site. Please see figure 5 flowchart for further details.


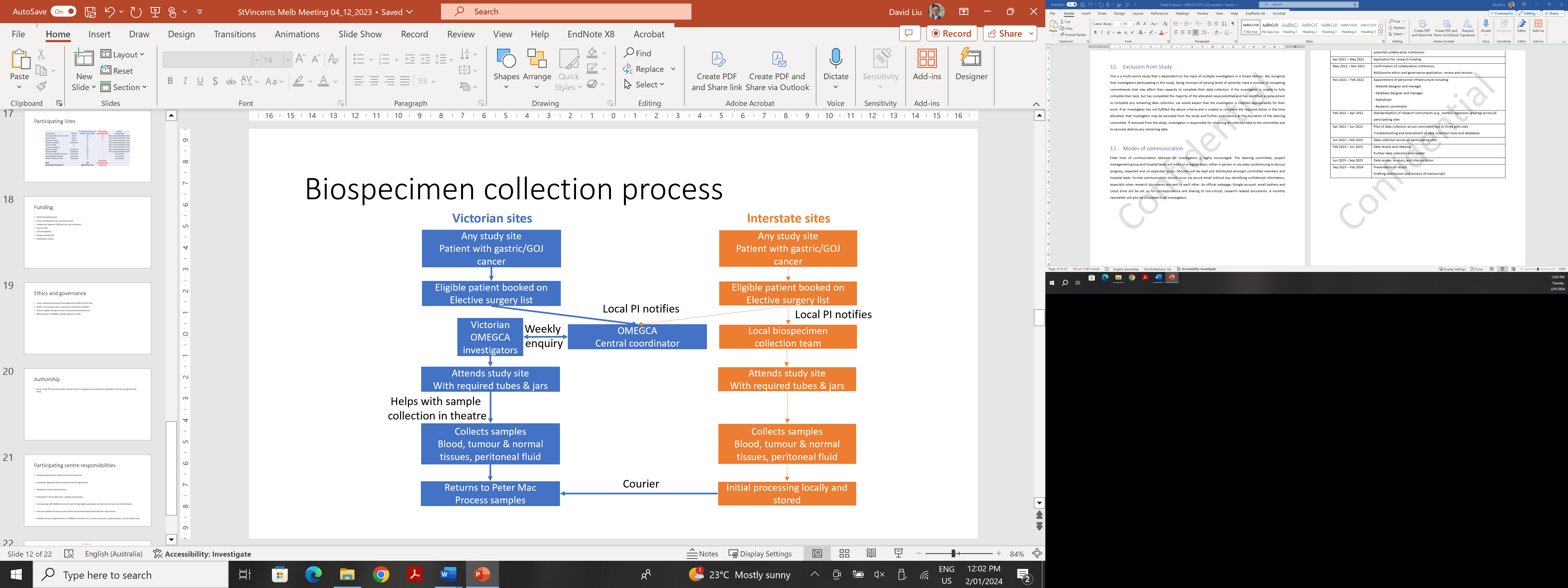


***Figure 5.*** *Flowchart of biospecimens collection process for Victorian and Interstate sites*

TYPES OF BIOSPECIMENS

| **Timepoint of collection**  **Biospecimens** | **At staging laparoscopy** | **At surgical resection** | **At post-surgery clinic** |
| --- | --- | --- | --- |
| **Peripheral blood** | 20-30 ml  Prior to skin incision | 20-30 ml  Prior to skin incision | 20-30 ml  (when possible) |
| **Primary tumour tissue** | 8-12 total endoscopic biopsies  4-6 in normal saline  4-6 in formalin | - | - |
| **Normal stomach tissue** | 4-6 endoscopic biopsies  in normal saline | - | - |
| **Normal oesophageal tissue** | 4-6 endoscopic biopsies  In normal saline | - | - |
| **Peritoneal fluid** | 150-250 ml of 500 ml 0.9% saline. 4 quadrants peritoneal lavage | 150-250 ml of 500ml 0.9% saline. 4 quadrant peritoneal lavage at start of surgery | - |

Please note the following

- Venepuncture, endoscopy, and peritoneal lavage are performed as standard-of-care at staging laparoscopy.
- Venepuncture and peritoneal access (either laparoscopic or open surgery) are performed as standard-of-care at surgical resection.
- Venepuncture is commonly performed at post-surgery outpatient review.
- Sampling 150-250 ml of peritoneal lavage fluid at staging laparoscopy does not impact standard cytological assessment.
- Please see section 5b below for further information.

PROCESSING OF BIOSPECIMENS:

| **Samples**  **Time period** | **Blood** | **Tissue fresh**  **(Tissue in saline)** | **Tissue fixed**  **(Tumour in formalin)** | **Peritoneal fluid** |
| --- | --- | --- | --- | --- |
| **From collection to lab** |  |  |  |  |
| Transport temperature | Room temperature | On ice | Room temperature | On ice |
| Transport medium | 10 ml Streck tubes x2 OR  10 ml EDTA tubes x2 | Normal saline | Formalin | In clean container |
| Ideal timeframe to lab | EDTA tubes: if processing <4 hr of collection  Streck tube: if processing 4 hr - 10 days post collection | <2 hrs of collection | Together with other specimens | Same day processing as collection |
| **Initial lab processing** |  |  |  |  |
| Procedure | As per laboratory manual | As per laboratory manual | As per laboratory manual | As per laboratory manual |
| Ideal timeframe | EDTA tubes: <4 hr post collection  Streck tube: <10 days post collection | <2 hrs of collection. Snap freeze in liquid nitrogen | Transfer into 70% ethanol, 1-3 days after being in formalin | Same day processing as collection |
| **Sample storage** |  |  |  |  |
| Storage temperature | -80 C | -80 C | Room temperature | -80 C |
| Storage location | Freezer | Freezer | 70% ethanol | Freezer |
| **Courier**  **(For interstate sites)** | In dry ice | In dry ice | Room temperature | In dry ice |

CLINICAL DATA COLLECTION:

Entry of clinical, radiological (CT and PET reports), endoscopic (reports), and histopathological (reports) data will be performed by local site research nurses/study coordinators, or designated surgical and medical oncology fellows already employed in clinical roles within each centre. For patients who fail to attend in-person follow-up, local clinicians and PIs at each site may attempt to contact these patients, as per standard-of-care, via telephone or videoconferencing to attain clinical follow-up and outcome data. Various quality assurance measures will be applied to minimise inter-observer discrepancies in clinical data entry. These measures will include:

- Data entry training
- Standardised electronic data collection tool
- In program prompting
- Real-time data entry support
- 10% random auditing of entered data as per the data management plan
- Complete cleaning to ensure accuracy before final analysis, as per the data management plan

GENOMIC AND METHYLOMIC ANALYSIS:

DNA from peritoneal lavage fluid, plasma, and primary cancer tissue will be extracted. These samples will be analysed using: 1) Tumour-informed genomic analysis, and 2) Tumour-agnostic methylomic analysis.

*Tumour-informed genomic analysis*: Tumour tissue and peritoneal fluid will be sent to the Johns Hopkins University Hospital, Kimmel Cancer Centre, USA (Collaborator: Prof Bert Vogelstein), and plasma DNA will be sent to Haystack Oncology^Pty Ltd^, USA, both for genomics analysis. Tumour specific mutations will be identified via whole exome sequencing of the primary tumour DNA and screened against blood derived buffy coat DNA to exclude germ-line mutations. This will inform targeted mutation assays to be performed on DNA derived from peritoneal lavage fluid and plasma, resulting in a binary output of positive or negative for the presence of ptDNA and ctDNA, respectively.

*Tumour-agnostic methylomic analysis*: Tumour tissue, plasma and peritoneal fluid will undergo methylomic sequencing and analysis at the Peter MaCallum Cancer Centre, through the Translational Genomics Core Facility (Collaborator: Dr Stephen Wong). Characteristic tumour methylation markers, consisting of the top differentially methylated regions between gastroesophageal cancer vs. adjacent normal tissue^22^ will be quantified using Methyl-seq.^23^ DNA derived from peritoneal lavage fluid will be sequenced to identify these methylation markers, resulting in a binary output of positive or negative for the presence of ptDNA.

SURVIVAL ENDPOINT DETECTION:

All patients will undergo routine clinical, endoscopic and imaging surveillance as per their local hospital guidelines and practice. This is typically as per the following timelines:

| **Time post-surgery** | **0-1 year** | **1-3 year** | **3-5 year** |
| --- | --- | --- | --- |
| **Clinical** | 3 monthly reviews | 6 monthly reviews | Annual reviews |
| **Imaging CT and/or PET** | At 1 year | Annually | Annually |
| **Endoscopy** | At 1 year | At 2^nd^ and 3^rd^ year | - |

CLINICAL ENCOUNTERS & SPECIMEN COLLECTION:

The table below highlights study-specific clinical encounters of patients, biospecimen collection, and data entry timepoints.

| **Clinical Schedules** | **Timing (location) of assessments/procedures**  **Assessment/Procedure** | **Pre-surgical visits**  **(Clinic)** | **Staging Laparoscopy**  **(Theatre)** | **Surgical Resection**  **(Theatre)** | **Post-operative**  **follow-up**  **(Clinic)** | **Long-term follow-up**  **1-5 years**  **(Clinic)#** |
| --- | --- | --- | --- | --- | --- | --- |
|  | **Informed Consent** | **X** | Reaffirm |  |  |  |
|  | **Demographic data** | **X** |  |  |  |  |
|  | **Clinical data** | **X** | **X** | **X** | **X** | **X** |
|  | **Histopathological data** |  |  |  | **X** |  |
|  | **CT and PET data** |  |  |  | **X** | **X** |
|  | **Endoscopy data** |  |  |  | **X** | **X** |
|  | **Blood collection** |  | **X** | **X** | **X^** |  |
|  | **Tissue biopsies collection** |  | **X** |  |  |  |
|  | **Peritoneal lavage collection** |  | **X** | **X*** |  |  |
|  | **Survival/recurrence data** |  |  |  |  | **X** |

**X:** designates when such data is generated and is captured as part of this study. ***** Only applicable to participants who undergo neoadjuvant therapy. **^** Where possible. **#** timepoints as per SURVIVAL ENDPOINT DETECTION table above.

### Standard Care and Additional to Standard Care Procedures

OMEGCA is a prospective cohort study. Standard-of-care (i.e., disease staging, treatment, and follow-up) will not be affected or altered by research encounters and collection of specimens. Biospecimen collections which are additional to standard-of-care include blood collections at all clinical timepoints, biopsy of tumour and peritoneal lavage fluid collection at surgical resection.

**Standard-of-care procedures**

| **Clinical Schedules** | **Timing (location) of assessments/procedures**  **Assessment/Procedure** | **Pre-surgical visits**  **(Clinic)** | **Staging Laparoscopy**  **(Theatre)** | **Surgical Resection**  **(Theatre)** | **Post-operative**  **follow-up**  **(Clinic)** | **Long-term follow-up**  **1-5 years**  **(Clinic)#** |
| --- | --- | --- | --- | --- | --- | --- |
|  | **Demographic history** | **X** |  |  |  |  |
|  | **Clinical history** | **X** | **X** | **X** | **X** | **X** |
|  | **Histopathology** |  |  |  | **X** |  |
|  | **CT and PET** |  |  |  | **X** | **X** |
|  | **Endoscopy follow-up** |  |  |  | **X** | **X** |
|  | **Venepuncture, Blood collection** |  | **X** | **X** | **X** |  |
|  | **Endoscopy and tissue biopsies** |  | **X** |  |  |  |
|  | **Peritoneal lavage** |  | **X** |  |  |  |
|  | **Survival and recurrence follow-up** |  |  |  |  | **X** |

**Addition to standard-of-care procedures**

| **Clinical Schedules** | **Timing (location) of assessments/procedures**  **Assessment/Procedure** | **Staging Laparoscopy**  **(Theatre)** | **Surgical Resection**  **(Theatre)** | **Post-operative**  **follow-up**  **(Clinic)** |
| --- | --- | --- | --- | --- |
|  | **Research blood collection** | **X** | **X** | **X** |
|  | **Research normal and tumour tissue biopsies** | **X** |  |  |
|  | **Research peritoneal lavage collection** | **X** | **X*** |  |

* Only applicable to participants who undergo neoadjuvant therapy.

- *Research blood collection*: 20-30 ml of peripheral blood will be collected prior to skin incision or in outpatient setting.
- *Research normal tissues biopsies*: 4-6 endoscopic biopsies of normal gastric and oesophageal mucosa
- *Research primary tumour biopsies*: 8-12 endoscopic biopsies of primary tumour
- *Peritoneal lavage fluid collection*: 150-250 ml of 500 ml 0.9% saline following 4 quadrant lavage of the abdominal cavity

STANDARDISING PERITONEAL LAVAGE COLLECTION AT STAGING LAPAROSCOPY:

During staging laparoscopy, there is currently NO standardised practice of performing peritoneal lavage. Between centres, there is variability with respect to the volume of saline (typically range from 0 ml to 1 L) applied and the quadrants of the abdomen (typically range from upper 2 quadrants to 4 quadrants) that are washed. Additionally, the pathology department within each hospital typically process and analyse variable amounts of submitted peritoneal lavage fluid. The remainder of this fluid is discarded. Therefore, in this study, we will standardise peritoneal lavage at staging laparoscopy to 500 ml 0.9% saline wash of all 4 quadrants of the abdomen. From this 300 ml will be submitted to anatomical pathology for routine processing and cytological analysis, and 200 ml will be used for ptDNA detection and analysis.

STANDARDISING PERITONEAL LAVAGE COLLECTION AT SURGICAL RESECTION:

For patients who have undergone neoadjuvant therapy, a further peritoneal lavage will be performed at surgical resection. Peritoneal lavage will be performed as soon as possible after achieving peritoneal access (either laparoscopically or via laparotomy). This will involve 500 ml 0.9% saline wash of all 4 quadrants of the abdomen. From this 200 ml will be used for ptDNA detection and analysis. The remaining 300 ml will be discarded.

## **Study Population**

### Recruitment Procedure

All patients referred to the listed health networks with gastroesophageal cancer for curative-intent treatment will be considered for recruitment. All patients who fulfill study eligibility criteria (section 6b) will be able to participate in this study. Potential study participants will be identified through hospital outpatient clinics, approached by the treating clinician, local PI, and/or research fellow, and a verbal description of the study together with the patient information consent form will be provided. Professional interpreters may be used where appropriate to translate the discussion. Identification and recruitment of patients, as well as data collection will be performed by health professionals that are part of the clinical team who provide care for patients.

### Inclusion Criteria

- All patients ≥18 years-of-age at time of signing consent
- Have a histologically confirmed diagnosis of one of the following:
  - Gastric adenocarcinoma
  - Gastroesophageal junction adenocarcinoma
- Is undergoing staging laparoscopy and peritoneal lavage cytology
- Provides informed consent

### Exclusion Criteria

- Strictly oesophageal cancer
- Distant organ disease on CT and PET/CT
- Non-regional nodal disease on CT and PET/CT
- Peritoneal carcinomatosis on CT and PET/CT
- Undergoing emergency surgery
- Performance status ECOG ≥3 (Appendix 1.1)
- Palliative-intent treatment

### Consent

Patients who agree to participate in the project will be consented by a member of the treating team, local PI or local sub-PI affiliated with this project. The participant’s competence and capacity to consent to the study will be assessed by one of these members. Any patient who is not able to give valid, informed consent for themselves will not be considered for inclusion. Patients will be asked to consent to the collection of biospecimens and associated data as detailed in section 5a. They will also be asked to consent to the storage and future use of these samples and data. All procedures will be conducted in accordance with the Declaration of Helsinki. Please refer to the Patient Information and Consent form for more details.

The consent process will occur significantly ahead of any clinical procedure. The clinician/investigator will allow time to answer any questions from the patient regarding the project and provide the patient time to consider their involvement. The clinician will also emphasize that the standard of care will not be altered in any way, regardless of the patient’s consent. On the morning of staging laparoscopy/endoscopy and definitive surgery, the project will be discussed again with participants. They will be given the opportunity to ask questions. For participants who have already given consent, local investigators will verbally re-affirm their consent prior to the start of their procedure and the collection of samples. For potential participants who have not yet signed the consent form, they will be asked if they wish to sign the form before their procedure. Patients are under no obligations to sign the consent form, and for those who have signed and then changed their mind, they may withdraw consent at any time (See below 7b, Handling of withdrawals for further detail).

The clinician/investigator will facilitate signing of the PICF and provide the participant with a copy. A copy of the PICF will be added to the participants medical records. The original PICF will be digitised and stored locally in keeping with each institution’s practices. The hardcopy will be kept in a locked filing cabinet in a locked office.

For any prospective participant recruited under OMEGCA, individual explicit consent will be sought as outline above. For participants from Project No 18/211, ‘*Improving outcomes in gastroesophageal cancer*’, consent has already been provided for the collection and use of their biospecimens and clinical data under HREC/44873/PMCC-2018. In particular, this includes DNA analysis for biomarker studies. Moreover, this consent included the use of their biospecimens and clinical data in other/future projects without the need for repeated consenting. Furthermore, it is impractical to obtain repeated consents from this cohort as, due to the nature of the disease being studied, a considerable number of these patients may be deceased. Additionally, the required clinical data have already been collected as part of routine care and will be de-identified, carrying negligible risk to participant confidentiality. It is therefore reasonable to assume that there is no likely reason for thinking that participants would not have consented if circumstances had allowed for them to be asked.

# **Participant Safety and Withdrawal**

### Risk Management and Safety

POTENTIAL RISKS – BLOOD COLLECTION:

Having a blood sample taken may cause some discomfort, bruising, minor infection or bleeding. All blood samples will be taken by trained personnel who are qualified to manage any such problems. However, in most cases, taking blood for this study will not involve any additional discomfort or risk since wherever possible this will be collected through the cannula which is routinely inserted as part of the standard endoscopy/surgery procedure.

POTENTIAL RISKS – COLLECTION OF PERITONEAL FLUID:

There is no additional risk or burden to the patient from collecting the waste fluid from their peritoneal wash that is performed as part of their standard of care.

POTENTIAL RISKS – TISSUE BIOPSIES:

Taking extra biopsies has minimal risk of harm as the participants are already having an endoscopy and the biopsies themselves are very small. However, taking biopsies for research means the endoscopy/laparoscopy will take a few minutes longer.

POTENTIAL RISKS – GENETIC INFORMATION:

There is a very small possibility that the research may result in new genetic information about a participant’s specific disease or their risk of getting other conditions. If research findings are made that may have significant implications for a participant or their family, we will submit a full report to the Peter MacCallum Human Research Ethics Committee and the appropriate established procedures will be followed. Participants will be given the choice as to whether or not they wish to know of any important results that has significant implications for them or their family. We will also ask participants if they want the information to be given to a member of their family if they cannot be contacted directly. We will not give any information about the participant to members of their family without their permission. It is possible that in the future genetic information from this study may affect a participant or their family when taking out a new life or income protection insurance policy or increase an existing life or income protection insurance policy. We will not pass on genetic information about a participant to anyone, including family members, without their written permission unless required to by law.

OVERALL RISK MITIGATION STRATEGIES:

To ensure smooth and safe collection of patients’ specimens, all specimen samples (blood, tissue biopsies and peritoneal lavage) will be collected by qualified clinicians who are well informed of the collection protocol. Blood collection at staging laparoscopy and surgical resection will be done by anaesthetists involved in the patient’s care. Tissue biopsies and peritoneal lavage will be collected by a member of the surgical team. Overall, in the unlikely event that something does arise that may significantly impact a patient’s welfare, we will follow established procedures and submit a full report to the Human Research Ethics Committee to seek advice on the most appropriate course of action.

### Handling of Withdrawals

Patients may choose to withdraw from the study at any time at their own volition. The following scenarios may apply:

- Withdrawal from study completely
- Withdrawal from further biospecimen collection with already collected biospecimen and clinical data
- Withdrawal from further clinical data collection with already collected biospecimen and clinical data

*Withdrawal from study completely*: Should a patient withdraw consent from further participation in this study, they will undergo routine clinical care without any additional biospecimen collection and all clinical data collection relating to the patient will cease. Wherever possible, all collected and analysed data will be electronically and/or manually deleted from our study records. All collected tissues and fluid will be disposed of appropriately. This will not impact on our ability to use samples and data obtained from other participants and therefore will not have any overall adverse impact on our project. Occasionally, it may not be possible to destroy material that has already been processed.

*Withdrawal from further biospecimen collection with already collected biospecimen and clinical data*: Should a patient choose to remain in the study, they will undergo routine clinical care without any additional specimen collection. However, the collection of their clinical data will continue, and the already collected specimen will be included in the final analysis.

*Withdrawal from further clinical data collection with already collected biospecimen and clinical data*: Should a patient choose to remain in the study, they will undergo routine clinical care without any additional clinical data collection. However, where appropriate, the already collected specimen and clinical data will be included in appropriate analysis e.g. initial validation experiments.

### Replacements

Due to the relatively low sample size required to address our primary endpoint, we plan to replace all withdrawn participants through additional recruitment to ensure that this study meets the sample size calculated in 8a.

# **Statistical Methods**

### Sample Size Estimation & Justification & Power Calculation

POWER JUSTIFICATION:

OMEGCA will have 4 observational cohorts as listed below:

- Group 1: Peritoneal lavage cytology-negative & ptDNA-negative
- Group 2: Peritoneal lavage cytology-negative & ptDNA-positive
- Group 3: Peritoneal lavage cytology-positive & ptDNA-negative
- Group 4: Peritoneal lavage cytology-positive & ptDNA-positive

However, as the primary objective of this study is to evaluate the clinical utility of ptDNA as a biomarker to predict the primary endpoint (2-year DFS) in gastroesophageal cancer patients with no apparent micro-metastatic disease in the peritoneum (using current ‘best practice’ staging methods), we have powered this study to compare groups 1 and 2, as these are the patients with *peritoneal lavage cytology-negative* disease. Accordingly, we deemed a 30% absolute DFS difference at 2 years post staging laparoscopy as clinically significant between groups 1 and 2. This is based on our hypothesis that patients who are ptDNA-positive should by definition have cytology-positive disease, even if this was not detected. The conservative estimate of 2-year DFS in patients who are cytology-positive versus cytology-negative is 40% versus 70%, respectively. Using our pilot data (Figure 3), we anticipate approximately 1 in 8 patients who is cytology-negative will be ptDNA-positive, this means an enrolment ratio of 7 to 1 in groups 1 and 2, respectively. To detect a 30% absolute difference in 2-year DFS between these two groups, we would need to enrol 200 patients with 175 patients in group 1 (ptDNA-negative arm) and 25 patients in group 2 (ptDNA-positive arm) to reach 80% statistical power at an alpha <0.05.

INTERIM ANALYSIS:

Based on centre volume, the number of participating sites, the number of patients already recruited on 18/211, and accounting for a 30% participant attrition rate, we anticipate to complete patient recruitment in 2 years. We will perform an interim analysis at 1 and 2 years after commencement of recruitment to ensure that the enrolment ratio between groups 1 (cytology-negative/ptDNA-negative) and 2 (cytology-negative/ptDNA-positive) is on-track (*Figure 4*). This will be measured by analysing the proportion of patients who are cytology-negative and ptDNA-positive. If the ratio of ptDNA-positive/cytology-negative is ≥10%, then recruitment of patients will continue to the target of 200. If this ratio is <10%, we will adjust our recruitment strategy accordingly. Additionally, we will perform an interim analysis at 3 years after commencement of patient recruitment (i.e. ≥1-year DFS data) to compare DFS between groups.

### Statistical Methods To Be Undertaken

FINAL ANALYSIS:

The clinical utility of ptDNA (ptDNA-positive vs ptDNA-negative), as determined by genomic and methylomic output, will be evaluated in consultation with our study biostatistician (Dr Darren Wong) using the following metrics: sensitivity, specificity, positive-predictive value, negative-predictive value, receiver operator characteristics, Kaplan Meier and Cox regression, against the primary and secondary endpoints. Co-variates in these analyses will be accounted for using hierarchical multi-variate logistic regression algorithms.

Additionally, exploratory analyses will be performed to:

- Compare ptDNA detection rate before (at time of PLC) and after (at time of surgical resection) neoadjuvant therapy.
- Compare ptDNA versus circulating tumour DNA (i.e. plasma) to predict sites and patterns of disease recurrence.
- Compare the cost-effectiveness of genomic vs. methylomic approaches to detect ptDNA to inform translation into clinical practice.
  - Using data collected including admission and discharge dates, length of stay, clinical services provided to each episode of care (e.g. CT, PET, laparoscopy, chemotherapy, radiotherapy, surgery etc.), outpatient follow-up, treating clinical units, estimated unit costs based on diagnosis-related group (DRG) and DRG descriptions, we will calculate the actual cost associated with each patient’s care along their cancer treatment journey. Then at a population level, this will be compared to a hypothetical projected cost based on whether ptDNA status changes clinical management, and whether this cost difference offsets the cost of a ptDNA assay.
- Curate and catalogue actionable molecular targets identified from whole exome sequencing of peritoneal wash fluid to inform future clinical trials.

These analyses will be performed using chi-square tests, Pearson correlations and other qualitative methodologies.

# **Storage of Blood and Tissue Samples**

## Details of where samples will be stored, and the type of consent for future use of samples

Collected tissue, blood and peritoneal fluid specimens will be transferred to the laboratory at Peter MacCallum Cancer Centre and stored securely. Specimens may undergo initial processing in the laboratory and then be kept in -80^o^C and/or -20^o^C freezers within the laboratory until further processing. Some specimens may be transferred to collaborative laboratories for sequencing under the associated Material Transfer Agreement. All specimens will be catalogued in a de-identified manner.

Any samples not used immediately will be stored indefinitely for future related research studies. Any future studies that use these samples will have to be approved by an appropriately constituted Human Research and Ethics Committee before they can be used. Participants who have consented to this project will not receive any notice of future uses of their information or samples for reasons outline in section 6d.

# **Data Security & Handling**

### Details of where records will be kept & How long will they be stored

CLINICAL, PET/CT, ENDOSCOPY AND HISTOPATHOLOGICAL DATA:

- Further information can be found in the associated data management plan. In summary:
- All data will be de-identified before entry into REDCap database. This database will be hosted at the Peter MacCallum Cancer Centre and governed by the hospital’s information technology and security processes. This includes appropriate best practices such as network firewalls, system and security monitoring and a two-factor authentication.
- REDCap also implements authentication to validate the identity of users that log in to the system. REDCap maintains an audit trail that logs user activity, including contextual information (e.g. the project or record being edited). Activities such as entering data, exporting data, modifying a field, running a report, or add/modifying a user, among a plethora of other activities are logged by REDCap. The logging record can be viewed by users who have appropriate privileges.
- REDCap access privileges will be managed and maintained by the coordinating principal investigators and study coordinator/project manager alongside Peter MacCallum Cancer Centre REDCap managers.
- The coordinating principal investigators, database manager, and statistician will have access to the entire database for database monitoring and analytical purposes.
- Principal investigators at each site can only view their own hospital’s data.
- Patient identifiers will be replaced with a unique study number. The site-specific master list of names and matching codes will be stored on password protected network at each participating site, with access to them only by staff directly involved with the project as determined by site principal investigator. This will enable re-identification should this situation arise. These site-specific master list will be destroyed once the project is closed. No identifiable information will be shared with collaborators outside the study investigators unless otherwise specified in an agreement or approved protocol.
- Data will be stored for at least 15 years after the completion of research activity.

PROCESSED GENOMIC AND METHYLOMIC DATA:

- This data will be stored on a password protected hard drive within the Clemons research laboratory hosted by a secured Peter MacCallum Cancer Centre server.
- This data will be linked to the REDcap clinical data by each participant’s unique study number.
- The coordinating principal investigators and designated sub-investigators will have access to these data.

### Confidentiality and Security

In addition to the details described above in section 10a. the following steps will also be undertaken to maintain the confidentiality of patients and their clinical data.

- Tissue and clinical data will be coded with a unique study number. The linker between the study number and patient identifier will be stored locally at each site. This will allow for the sample to be linked back to the participant’s medical records if required. In the case of samples collected externally (e.g. St Vincent’s Hospital, Monash Health etc.) the appropriate staff at these organisations will be contacted to extract data from the medical records.
- All data generated from this study will remain confidential and no published work will contain patient identifiers.
- Any publication or presentation that arise from this project will be presented as general cohort information with numbers and statistics. No individual data will be published or shared to ensure that identification of individual patients is not possible.
- All study-related personnel are bound by professional standards of patient information confidentiality and will work to protect patient confidentiality at all times.

# **Results, Outcomes & Future Plans**

### Results and reporting of outcomes

Study outcomes will be largely disseminated through traditional approaches such as peer-reviewed scientific literature, conference presentations and seminars. In any publication and/or presentation, information will be provided in such a way that individual participants cannot be identified, except with their permission. There is no formal plan to return results of this project to participants who provide tissue, blood samples or peritoneal fluid, and no individual results will be returned except in the rare case of incidental findings that may have an impact on the health of the participant or their family and the participant has consented to being contacted. This is because the planned research, by its very nature is experimental, and any new discoveries may take considerable time before they can be translated into clinically relevant results. Furthermore, this kind of research can take a long time and individual results are unlikely to have any significance except when pooled with data from other individuals. However, upon request, the Principal Investigators will provide a summary of the research findings to a participant.

### Additional studies

It is likely that through the course of the project, additional research questions may arise that require additional data to be collected either for all of the patients or for a specific subset. Such a scenario will involve the collection of data over and above that specifically detailed in this proposal. If clinicians desire to collect additional data, funding and ethical approval for this must be obtained separately. The collection, analysis and reporting of any additional data beyond that specifically detailed in this proposal will only occur with agreement of all individual site Principal Investigators, and only with ethical approval.

### Project closure processes

After 5 years of survival data has been collected on all patients, final outcome data analysis will be performed and all study data archived under the care of the coordinating principal investigators and study coordinator/project manager. All data will be kept in a de-identified, password protected format in the Clemon’s Research Laboratory for a further period of not less than 15 years before secure electronic file deletion.

# **Investigator’s responsibilities**

CO-COORDINATING PRINCIPAL INVESTIGATORS:

Dr. David Liu and A/Prof. Nicholas Clemons will provide oversight of the entire project. In these roles, they will be responsible for ensuring compliance with ICH-GCP, the National Statement on Ethical Conduct of Human Research, and Institutional Research Standard Operating Procedures (SOPs.)

Specifically, A/Prof. Clemons will oversee the laboratory research aspects of the project and be responsible for the follow up experiments including biospecimen processing and data analysis.

Dr. David Liu is responsible for the implementation of all human subjects’ research aspects of the project related to obtaining informed consent from participants, defining any protocolized requirements for biospecimen collection, and final data analysis. Dr. David Liu will serve as contact CPI for regulatory purposes and will assume responsibility for fiscal and administrative management including maintaining communication among PIs and key personnel through regular meetings. Dr. David Liu will be responsible for communication with funding bodies and submission of annual reports.

PROJECT MANAGEMENT GROUP:

In line with ICH-GCP, Aa core group of multidisciplinary experts who have overall responsibility for:

- Overall scientific content and integrity
- Project oversight and support
- Protocol design
- Study site identification and selection
- Ethics and governance application
- Signing data and material transfer agreements
- Web-based design
- REDCap database design, monitoring and management
- Project co-ordination including liaison with hospital leads
- Dissemination of OMEGCA documents and results
- Data handling
- Data analysis
- Results interpretation
- Preparation of research manuscripts
- Journal submission and correspondence

HOSPITAL LEADS/PRINCIPAL INVESTIGATORS:

A lead point of contact at each site (1-2 people, at least one must be a consultant) who has overall responsibility for:

- Providing site-specific oversight and support
- Site governance registration and facilitating signing of data/material transfer agreements
- Identification of eligible patients
- Appointing, registering and supporting data collectors
- Ensuring data integrity from that site
- Local dissemination of OMEGCA documents and results
- Contributing to results interpretation
- Reporting to the Project Management Group

LOCAL COLLABORATORS/DATA COLLECTORS:

A mini-team of 1-3 (excluding hospital leads/principal investigator) people who have the overall responsibility for:

- Reviewing and understanding study objectives and methodologies
- Performing site-specific chart review and data entry into REDCap for ~ 20-60 patients each
- Integrity of data collected
- Being available to assist with data cleaning, corrections and data review
- Reporting to site principal investigator(s)

Please note that, mini-team size at each site will be at the discretion of the hospital lead according to the caseload of each hospital. Minimum requirements for authorship for local collaborators on OMEGCA output include:

- Compliance with local audit approval processes and data governance policies.
- Active involvement in data collection that meets the criteria for inclusion within the OMEGCA dataset.
- Collaboration with the hospital lead to ensure that data are reported back to the Project Management Group.

# **Criteria for centre inclusion within OMEGCA**

- Obtain all appropriate local governance approvals for the conduct of OMEGCA.
- Obtain signoff for data and material transfer agreements.
- For Victorian sites: Coordinate with OMEGCA research team to identify eligible patients, timing of operating lists, and facilitate biospecimen collection.
- Successful completion of data collection for eligible patients meeting inclusion criteria
- >90% data completeness and >90% data accuracy has been achieved.
- All data for the period has been uploaded within the specified deadlines.

Please note if these criteria are not met, then the contributing mini-team and/or the centre may be removed from the dataset and authorship list (please contact the Project Management Group as soon as potential issues arise).

# **Authorship for publications**

Individuals will be recognized as authors if they meet authorship criteria based on Australian Code for the Responsible conduct of Research

# **Exclusion from study**

This is a multi-centre study that is dependent on the input of multiple investigators in a timely fashion. We recognise that investigators participating in this study, being clinicians of varying levels of seniority, have a number of competing commitments that may affect their capacity to complete their data collection. If the investigator is unable to fully complete their task, but has completed the majority of the allocated responsibilities and has identified a replacement to complete any remaining data collection, we would expect that the investigator is credited appropriately for their work. If an investigator has not fulfilled the above criteria and is unable to complete the required duties in the time allocated, that investigator may be excluded from the study and further publications at the discretion of the Project Management Group. If removed from the study, investigator is responsible for returning all collected data to the committee and to securely destroy any remaining data.

# **Modes of communication**

Clear lines of communication between all investigators are highly encouraged. The Project Management Group and hospital leads will meet on a regular basis, either in person or via video conferencing to discuss progress, expected and un-expected issues. Minutes will be kept and distributed amongst committee members and hospital leads. Formal communication should occur via secure email without any identifying confidential information, especially when research documents are sent to each other. An official webpage, email address and cloud drive will be set up for correspondence and sharing of non-clinical, research related documents. A monthly newsletter will also be circulated to all investigators.

# **References**

1. Cancer Australia. Stomach cancer statistics. https://www.canceraustralia.gov.au/cancer-types/stomach-cancer/statistics. accessed 21/07/2023.

2. Cancer Australia. Oesophageal cancer in Australia. https://www.canceraustralia.gov.au/cancer-types/oesophageal-cancer/statistics. accessed 21/07/2023.

3. Sung H, Ferlay J, Siegel RL, et al. Global Cancer Statistics 2020: GLOBOCAN Estimates of Incidence and Mortality Worldwide for 36 Cancers in 185 Countries. *CA Cancer J Clin* 2021; 71(3):209-249.

4. Rice TW, Patil DT, Blackstone EH. 8th edition AJCC/UICC staging of cancers of the esophagus and esophagogastric junction: application to clinical practice. *Ann Cardiothorac Surg* 2017; 6(2):119-130.

5. Cho JH, Kim SS. Peritoneal Carcinomatosis and Its Mimics: Review of CT Findings for Differential Diagnosis. *J Belg Soc Radiol* 2020; 104(1):8.

6. Leake PA, Cardoso R, Seevaratnam R, et al. A systematic review of the accuracy and utility of peritoneal cytology in patients with gastric cancer. *Gastric Cancer* 2012; 15 Suppl 1:S27-37.

7. Lemoine L, Sugarbaker P, Van der Speeten K. Pathophysiology of colorectal peritoneal carcinomatosis: Role of the peritoneum. *World J Gastroenterol* 2016; 22(34):7692-707.

8. Kelly RJ, Ajani JA, Kuzdzal J, et al. Adjuvant Nivolumab in Resected Esophageal or Gastroesophageal Junction Cancer. *N Engl J Med* 2021; 384(13):1191-1203.

9. Al-Batran SE, Homann N, Pauligk C, et al. Perioperative chemotherapy with fluorouracil plus leucovorin, oxaliplatin, and docetaxel versus fluorouracil or capecitabine plus cisplatin and epirubicin for locally advanced, resectable gastric or gastro-oesophageal junction adenocarcinoma (FLOT4): a randomised, phase 2/3 trial. *Lancet* 2019; 393(10184):1948-1957.

10. Nakauchi M, Vos EL, Carr RA, et al. Distinct Differences in Gastroesophageal Junction and Gastric Adenocarcinoma in 2194 Patients: In Memory of Rebecca A. Carr, February 24, 1988-January 19, 2021. *Ann Surg* 2023; 277(4):629-636.

11. Harada H, Soeno T, Nishizawa N, et al. Prospective study to validate the clinical utility of DNA diagnosis of peritoneal fluid cytology test in gastric cancer. *Cancer Sci* 2021; 112(4):1644-1654.

12. Hiraki M, Kitajima Y, Koga Y, et al. Aberrant gene methylation is a biomarker for the detection of cancer cells in peritoneal wash samples from advanced gastric cancer patients. *Ann Surg Oncol* 2011; 18(10):3013-9.

13. Hiraki M, Kitajima Y, Sato S, et al. Aberrant gene methylation in the peritoneal fluid is a risk factor predicting peritoneal recurrence in gastric cancer. *World J Gastroenterol* 2010; 16(3):330-8.

14. Ushiku H, Yamashita K, Ema A, et al. DNA diagnosis of peritoneal fluid cytology test by CDO1 promoter DNA hypermethylation in gastric cancer. *Gastric Cancer* 2017; 20(5):784-792.

15. Yu QM, Wang XB, Luo J, et al. CDH1 methylation in preoperative peritoneal washes is an independent prognostic factor for gastric cancer. *J Surg Oncol* 2012; 106(6):765-71.

16. Yukawa N, Yamada T, Aoyama T, et al. Tumor DNA in Peritoneal Lavage as a Novel Biomarker for Predicting Peritoneal Recurrence in Patients With Gastric Cancer. *Anticancer Res* 2023; 43(5):2069-2076.

17. Russo A, Li P, Strong VE. Differences in the multimodal treatment of gastric cancer: East versus west. *J Surg Oncol* 2017; 115(5):603-614.

18. Cabalag CS, Yates M, Corrales MB, et al. Potential Clinical Utility of a Targeted Circulating Tumor DNA Assay in Esophageal Adenocarcinoma. *Ann Surg* 2022; 276(2):e120-e126.

19. Wong SQ, Raleigh JM, Callahan J, et al. Circulating Tumor DNA Analysis and Functional Imaging Provide Complementary Approaches for Comprehensive Disease Monitoring in Metastatic Melanoma. *JCO Precis Oncol* 2017; 1:1-14.

20. Tie J, Cohen JD, Lahouel K, et al. Circulating Tumor DNA Analysis Guiding Adjuvant Therapy in Stage II Colon Cancer. *N Engl J Med* 2022; 386(24):2261-2272.

21. Liu DS, Stevens SG, Watson DI, et al. Optimal Timing of Perioperative Chemoprophylaxis in Patients With High Thromboembolic Risk Undergoing Major Abdominal Surgery: A Multicenter Cohort Study. *Ann Surg* 2023; 277(1):79-86.

22. Li D, Zhang L, Liu Y, et al. Specific DNA methylation markers in the diagnosis and prognosis of esophageal cancer. *Aging (Albany NY)* 2019; 11(23):11640-11658.

23. Vaisvila R, Ponnaluri VKC, Sun Z, et al. Enzymatic methyl sequencing detects DNA methylation at single-base resolution from picograms of DNA. *Genome Res* 2021; 31(7):1280-9.

# **Appendix 1 – ECOG performance scale**


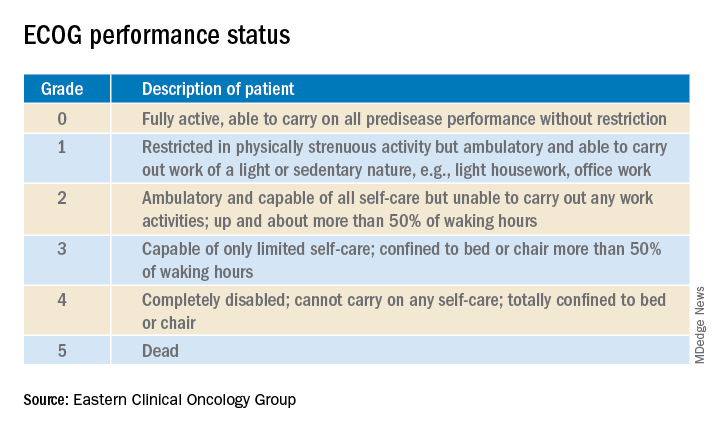

Supplement: S3 Supporting materials — (DOCX) [file pone.0318615.s003.docx]
